# Photo-engineered optoelectronic properties of indium tin oxide via reactive laser annealing

**DOI:** 10.1038/s41598-022-18883-5

**Published:** 2022-09-02

**Authors:** James Arthur Hillier, Panos Patsalas, Dimitrios Karfaridis, Sophie Camelio, Wayne Cranton, Alexei V. Nabok, Christopher J. Mellor, Demosthenes C. Koutsogeorgis, Nikolaos Kalfagiannis

**Affiliations:** 1 School of Science and Technology, Nottingham Trent University, Nottingham, NG11 8NS UK; 2 Department of Physics, Aristotle University of Thessaloniki, 54124 Thessaloniki, Greece; 3 UPR 3346, Institut Pprime, CNRS-Université de Poitiers, Poitiers, France; 4 Materials and Engineering Research Institute, Sheffield Hallam University, Sheffield, S1 1WB UK; 5 School of Physics and Astronomy, The University of Nottingham, Nottingham, NG7 2RD UK

**Keywords:** Materials science, Nanoscience and technology, Optics and photonics

## Abstract

Transparent conductive oxides are appealing materials for optoelectronic and plasmonic applications as, amongst other advantages, their properties can be modulated by engineering their defects. Optimisation of this adjustment is, however, a complex design problem. This work examined the modification of the carrier transport properties of sputtered tin-doped indium oxide (ITO) via laser annealing in reactive environments. We relate the optical modifications to the structural, compositional, and electronic properties to elucidate the precise mechanisms behind the reactive laser annealing (ReLA) process. For sufficiently high laser fluence, we reveal an ambient-dependent and purely compositional modulation of the carrier concentration of ITO thin films. Hereby, we demonstrate that ReLA utilises the precise energy delivery of photonic processing to enhance the carrier mobility and finely tune the carrier concentration without significantly affecting the crystal structure. Exploitation of this phenomena may enable one to selectively engineer the optoelectronic properties of ITO, promising an alternative to the exploration of new materials for optoelectronic and photonic applications.

## Introduction

Transparent conductive oxides (TCOs) are used in optoelectronic devices such as flat panel displays due to their transparency, refractory character, and their capability for dynamic tuning of their optoelectronic behaviour through application of electric fields via the Pockels effect^1–3^. An important additional asset of TCOs is the fact that they are CMOS compatible, unlike the noble metals, allowing for utilisation of well-matured manufacturing techniques and integration into current electronic devices^4^. Vitally, the optoelectronic properties of TCOs are sensitive to the fabrication techniques and parameters^5^. For example, adjustment of the dopant level of the sputtering target, deposition and post-growth processing conditions enables one to tailor the optoelectronic properties of TCOs towards specific device requirements^6^. Such modifications, for tin-doped indium oxide (ITO) in particular, have been achieved through thermal annealing (TA) in controlled environmental conditions^7–9^. This has been shown to improve the free carrier mobility and transparency by inducing crystallisation of the as grown amorphous film. TA has also been demonstrated to adjust the carrier concentration by probing the donor state variations, which result from Sn^4+^ ion substitutions and the oxygen vacancy concentration^10–14^. TA within an ambient environment of $5\%$ H_2_ in N_2_ increased the carrier concentration by creating oxygen vacancies while, conversely, an ambient environment of pure O_2_ reduced the carrier concentration by filling oxygen vacancies^7^. However, TA of thin films suffers from long dwell times and high thermal budget, making the process, cumbersome and unable to be utilised for films where the characteristics of the substrate must not be compromised (i.e., flexible electronic devices or manufactured chips with heat-sensitive components). Recent results have demonstrated the ability of laser annealing (LA) to overcome these limitations and offer an ultra-fast, scalable, and low thermal budget post-growth processing technique to enhance the crystallinity of TCOs^15–17^. LA operates through the application of a highly spatially and temporally localised energetic heating and offers an increased level of control over the manipulation of material properties^18^. This is achieved via access to an array of LA parameters such as pulse length, frequency, number of pulses, fluence and wavelength in addition to environmental parameters such as pressure and composition^18,19^.

In this work, we seek to synergise the advantages of LA (KrF, $\uplambda=248\;\mathrm{ nm}$) with the ability to probe the defects by controlling the reactive ambient. Specifically, we investigate two “extremes” of a highly oxidising ($100\mathrm{\%}$ O_2_) and a highly reducing ($5\mathrm{\%}$ H_2_ in N_2_) atmosphere during LA with a single pulse and a varied laser fluence ($25{-}125\;\mathrm{ mJ c}{\mathrm{m}}^{-2}$). We perform extensive characterisation utilising: four-point probe (4pp), Hall Effect, X-ray diffractometry (XRD), X-ray photon spectroscopy (XPS), transmission electron microscopy (TEM), energy-dispersive X-ray spectroscopy (EDX) and spectroscopic ellipsometry (SE) across a wide spectral range ($0.034{-}3.34\;\mathrm{ eV}$). We relate the optical properties of the processed films to the electronic, structural, and compositional laser-induced modifications in order to gain knowledge of the physical mechanisms behind LA in reactive gas environments. We reveal a purely compositional, ambient-dependent modification of the carrier transport properties of ITO, demonstrating that “reactive LA” (ReLA) can enhance the carrier mobility and tune the carrier concentration without significantly affecting the crystal structure. Further exploitation opens a pathway to selectively engineer the defects of ITO.

## Results and discussion

### Optoelectronic properties of the ReLA-ITO films

**Figure 1:**
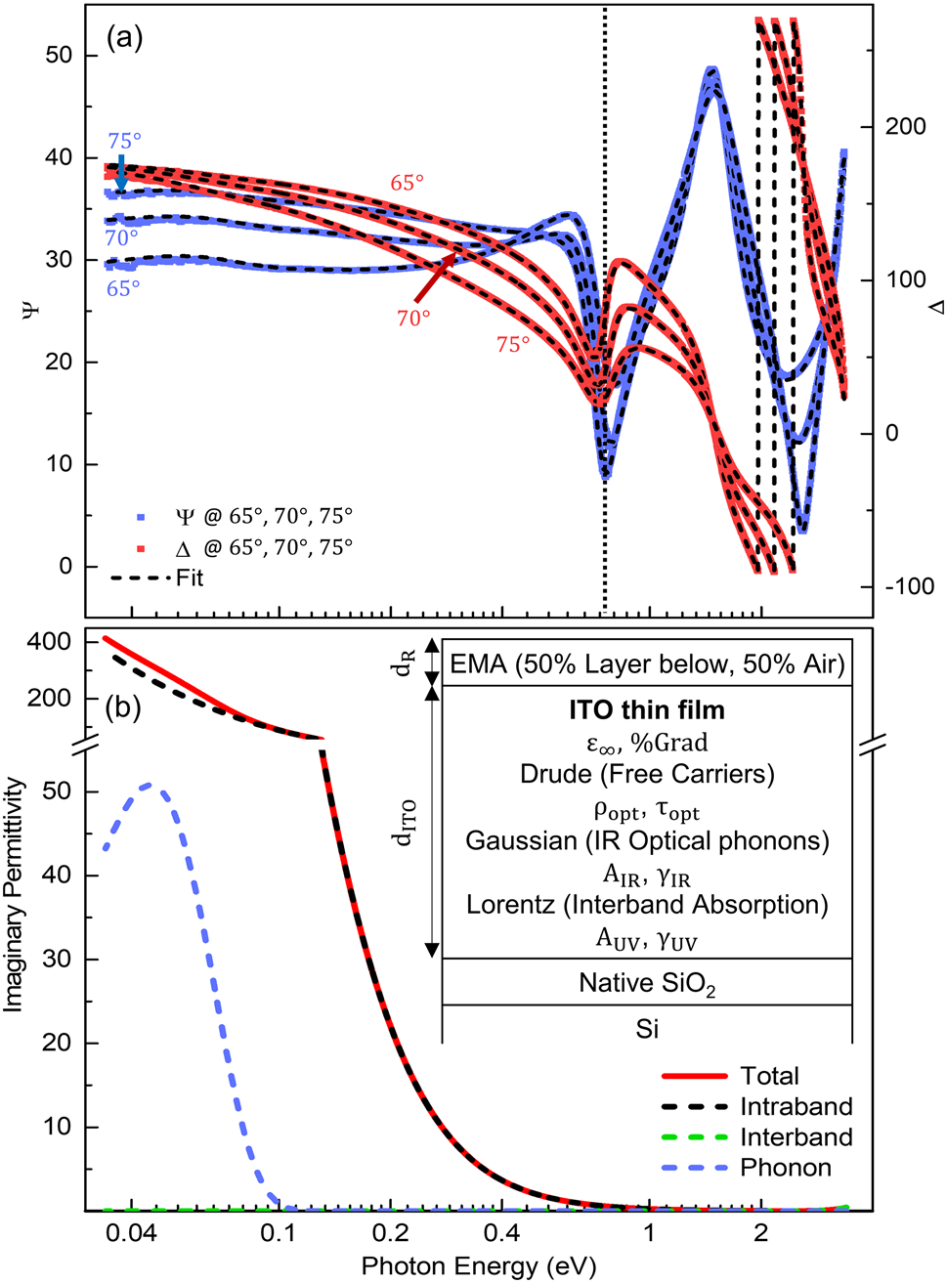
Ellipsometric measurement and as-fit permittivity of the seed ITO thin film. (**a**) Measured $\Psi (\mathrm{E})$ (blue squares) and $\Delta (\mathrm{E})$ (red squares) at $65^\circ$, $70^\circ$, and $70^\circ$ for the higher resistivity seed ITO thin film alongside their corresponding fits (dashed black lines). (**b**) The fitted imaginary permittivity of the seed ITO thin film (solid red line), where the individual components of the imaginary permittivity for the free carriers, interband transitions and phonon mode are represented by the dashed black, blue and green dashed lines, respectively. A logarithmic scale in the x-axis is used in (**a,b**) to exaggerate the IR spectral region. The vertical dotted black line in (**a**) presents the cut-off between the spectral ranges of the two IR and NIR-VIS ellipsometers. The inset in (**b**) presents a schematic of the geometric and parametrised optoelectronic model.

**Figure 2:**
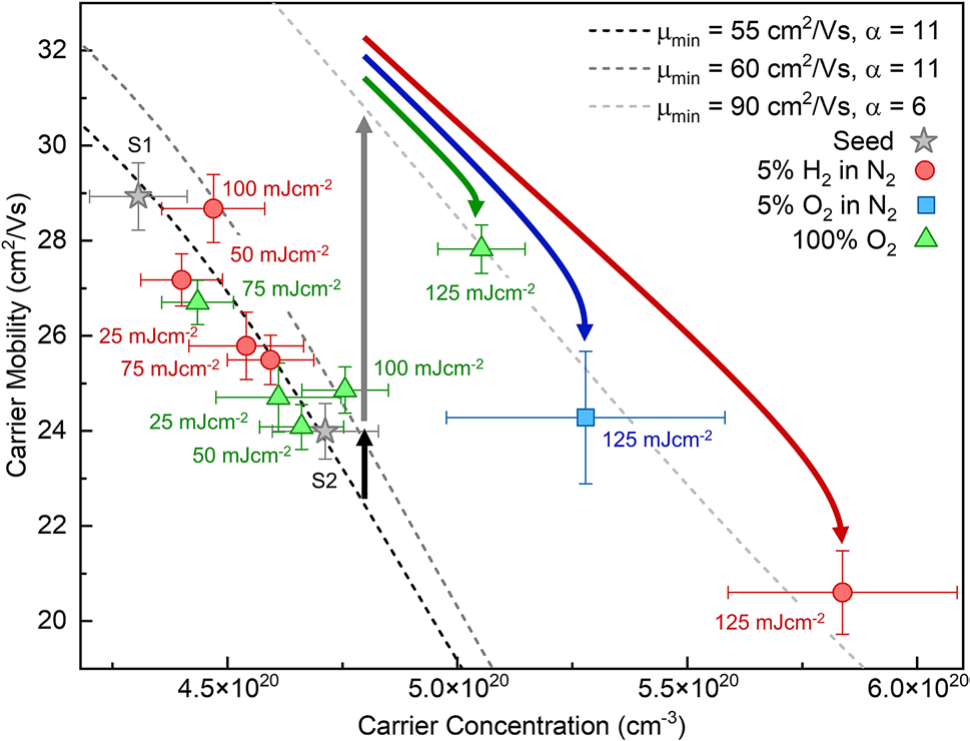
Optoelectronic properties of all seed and laser processed ITO thin films. “Optical” carrier concentration, ${\mathrm{N}}_{\mathrm{opt}}$, and mobility, ${\upmu }_{\mathrm{opt}}$, of the seed ITO thin films (grey stars) and those subject to single-pulse ReLA at $25{-}125\; {\mathrm{mJ cm}}^{-2}$ in $5\mathrm{\%}$ H_2_ in N_2_ (red circles) and $100\mathrm{\%}$ O_2_ (green triangles). We also present the sample processed with $125 \; {\mathrm{mJ cm}}^{-2}$ in $5\mathrm{\%}$ O_2_ in N_2_ with the blue square. The dashed lines indicate the theoretical trend of $\upmu (\mathrm{N})$ considering various scattering mechanisms. The parameters of $\upmu (\mathrm{N})$ that are related to ionised cluster scattering (${\upmu }_{\mathrm{min}}$ and $\mathrm{\alpha }$) were fitted to the seed materials and those subject to ReLA at below $100 \; {\mathrm{mJ cm}}^{-2}$ (black dashed line), at $100 \; {\mathrm{mJ cm}}^{-2}$ (dark grey dashed line) and at $125 \; {\mathrm{mJ cm}}^{-2}$ (light grey dashed line). The black and grey arrows indicate the mobility-only transition to the next cluster and the red, blue, and green arrows indicate the ambient dependent transition to higher ${\mathrm{N}}_{\mathrm{opt}}$ after ReLA at $125 \; {\mathrm{mJ cm}}^{-2}$ in $5\mathrm{\%}$ H_2_ in N_2_, $5\mathrm{\%}$ O_2_ in N_2_ and $100\mathrm{\%}$ O_2_, respectively.

In order to reveal the properties of the ITO films subjected to ReLA we firstly examine the optoelectronic properties (i.e., the complex permittivity), which define the key characteristics of TCOs that are associated with most applications^20,21^. Figure 1a shows the measured ellipsometric angles $\Psi (\mathrm{E})$ (blue squares) and $\Delta (\mathrm{E})$ (red squares) , measured at $65^\circ$, $70^\circ$, and $75^\circ$, of an indicative seed ITO thin film. $\Psi (\mathrm{E})$, $\Delta (\mathrm{E})$, ${\mathrm{T}}_{\mathrm{IR}}(\mathrm{E})$*,* and ${\mathrm{R}}_{\mathrm{VIS}}(\mathrm{E})$ for all samples can be found in the Supplementary, section B. For all samples, there is a perfect overlap between the IR and near-IR-visible (NIR-VIS) measurements. Therefore, we fit the total combined spectra, with ${\mathrm{T}}_{\mathrm{IR}}(\mathrm{E})$ and ${\mathrm{R}}_{\mathrm{VIS}}(\mathrm{E})$ appended to the data. By fitting all measurements simultaneously, we reduce the correlation between the fitting parameters and thus we improve the confidence in the uniqueness and physical reality of the extracted values. The best fit across the entire spectral range is presented with dashed black lines in Fig. 1a. To fit the measured $\Psi (\mathrm{E})$ and $\Delta (\mathrm{E})$ to the seed and laser processed ITO, we apply a geometric model that was derived from the architecture of the sample (ITO/$2.63\;\mathrm{ nm}$-SiO_2_/Si; presented schematically in Fig. 1b). To improve the fit, we follow the suggestions of previous reports to include a layer of surface roughness, described by an effective medium approximation (EMA) of $50{\%}$ ITO and $50{\%}$ void, and incorporate a simple gradient of the permittivity across the depth of the ITO film^22,23^. The optical model describes $\widetilde{\upvarepsilon }(\mathrm{E})$ for each layer. The imaginary permittivity of the ITO layer, ${\upvarepsilon }_{2}\left(\mathrm{E}\right)$, is described by a summation of multiple oscillators (Eq. 1), which are described further in the Supplementary, section C**.**

1 $$\widetilde{\upvarepsilon }\left(\mathrm{E}\right)={\upvarepsilon }_{\infty }+\frac{-{\mathrm{\hslash }}^{2}}{{\upvarepsilon }_{\mathrm{o}}{\uprho }_{\mathrm{opt}}\left({\uptau }_{\mathrm{opt}}{\mathrm{E}}^{2}+\mathrm{i\hslash E}\right)}+{\sum }_{\mathrm{n}=1}^{\mathrm{N}}{\widetilde{\upvarepsilon }}_{\mathrm{n}}\left(\mathrm{E}\right)$$

where ℏ is Plank’s constant, ${\upvarepsilon }_{\mathrm{o}}$ is the vacuum permittivity, ${\uprho }_{\mathrm{opt}}$ is the optical resistivity, and ${\uptau }_{\mathrm{opt}}$ is the scattering time. The third term summates multiple oscillators that describe phonon, defect and/or interband absorption. The real permittivity, ${\upvarepsilon }_{1}\left(\mathrm{E}\right)$, is calculated from a Kramers–Kronig transformation of ${\upvarepsilon }_{2}\left(\mathrm{E}\right)$ with a background contribution to the permittivity, ${\varepsilon }_{\infty }$^24^. By fitting the permittivity and geometry (thickness and roughness) of the ITO layer to the measurements, we extract the unknown variables in our model (e.g., thickness, ${\mathrm{d}}_{\mathrm{ITO}}$, surface roughness, ${\mathrm{d}}_{\mathrm{R}}$, “optical” resistivity, ${\uprho }_{\mathrm{opt}},$ and “optical” scattering time, ${\uptau }_{\mathrm{opt}}$). The designation “optical” refers to the fact that the parameter is defined in relation to an alternating driving electromagnetic field instead of by a static field as is the case of 4 pp and Hall effect measurements. The “optical” free carrier concentration, ${\mathrm{N}}_{\mathrm{opt}}$, and mobility, ${\upmu }_{\mathrm{opt}}$, are calculated from ${\uprho }_{\mathrm{opt}}$ and ${\uptau }_{\mathrm{opt}}$ via Eqs. (2 and 3).

2 $${\upmu }_{\mathrm{opt}}=\frac{{\uptau }_{\mathrm{opt}}\mathrm{e}}{{\mathrm{m}}_{\mathrm{e}}^{*}}$$

3 $$\mathrm{N}=\frac{{\mathrm{m}}_{\mathrm{e}}^{*}}{{\uprho }_{\mathrm{opt}}{\mathrm{e}}^{2}{\uptau }_{\mathrm{opt}}}$$

where $\mathrm{e}$ is the electron charge and ${\mathrm{m}}_{\mathrm{e}}^{*}={\mathrm{m}}^{*}{\mathrm{m}}_{\mathrm{e}}$ (${\mathrm{m}}^{*}$ is the effective mass ratio and ${\mathrm{m}}_{\mathrm{e}}$ is the electron mass). The set of fitting parameters and independently measured properties for all ITO thin films are summarised in Supplementary Tables S1 and S2. The effective electron mass, ${\mathrm{m}}_{\mathrm{e}}^{*}$, is determined for each sample by considering an increased non-parabolicity in the conduction band due to the free carrier population^25^. This process required independent measurement of the “Hall” carrier concentration, ${\mathrm{N}}_{\mathrm{Hall}}$, via Hall Effect and is outlined further in a previous publication^26^. $\mathrm{N}$ and $\upmu$ are linked through the influence of scattering mechanisms. The individual mechanisms that comprise the mobility dependence to the carrier concentration, $\upmu (\mathrm{N})$, have been described for the case of ITO by Ellmer et al*.*^27,28^. Therefore, to reveal the full picture of the transport properties of each seed and laser processed film, we present ${\upmu }_{\mathrm{opt}}$ against ${\mathrm{N}}_{\mathrm{opt}}$ and for the seed ITO thin films (grey stars) and those subject to single-pulse ReLA at $25{-}125\; {\mathrm{mJ cm}}^{-2}$ in $5\mathrm{\%}$ H_2_ in N_2_ (red circles), $5\mathrm{\%}$ O_2_ in N_2_ (blue square), and $100\mathrm{\%}$ O_2_ (green triangles) in Fig. 2. The labels indicate the fluence used during laser processing. Three distinct trends of $\upmu (\mathrm{N})$ are fit to individual “clusters” of the films: $25{-}75\; {\mathrm{mJ cm}}^{-2}$, $100\; {\mathrm{mJ cm}}^{-2}$, and $125\; {\mathrm{mJ cm}}^{-2}$, and are indicated in Fig. 2 with the black, dark grey, and light grey dashed lines, respectively. The “scattering equation” that defines $\upmu \left(\mathrm{N}\right)$ is expressed fully elsewhere^27,28^.

Consequently, we observe that the clusters relate to groups of samples where the scattering mechanisms that influence ${\upmu }_{\mathrm{opt}}$ in relation to $\mathrm{N}$ are identical. When $\mathrm{N}>{10}^{20} \;\mathrm{ c}{\mathrm{m}}^{-3}$, the dominant scattering mechanism of ITO is ionised cluster scattering (ICS)^28^. Within the small range of $\mathrm{N}$ studied, only the parameters for the ionised impurity scattering (IIS) diminished mobility, ${\upmu }_{\mathrm{min}}$, and the coefficient of ICS, ${\mathrm{\alpha }}_{\mathrm{ICS}}$, matter (due to Matthiessen’s rule). ${\mathrm{\alpha }}_{\mathrm{ICS}}$ depicts the scattering efficiency of the ionised clusters and, within the small range of N studied here, can be perceived as the negative gradient of $\upmu (\mathrm{N})$ (where ${\upmu }_{\mathrm{min}}$ represents the intercept). Thus, these are set as free parameters when fitting $\upmu (\mathrm{N})$ to each cluster. All other parameters are fixed to those found by examining a larger range of N^26,27^. The first cluster comprises the two seed films and those annealed in either $5{\%}$ H_2_ in N_2_ (red circles) and $100{\%}$ O_2_ (green triangles) at lower fluences ($25{-}75 \; \mathrm{ mJ c}{\mathrm{m}}^{-2}$). The two seed films cover the range of ${\uprho }_{\mathrm{opt}}$ demonstrated by the cluster of samples taken from the seed ITO film (see the Supplementary, section A). The films subject to ReLA at $25{-}75 \; \mathrm{ mJc}{\mathrm{m}}^{-2}$ lie along the theoretical trend of $\upmu (\mathrm{N})$, where ${\upmu }_{\mathrm{min}}=55 \; {\mathrm{cm}}^{2}/\mathrm{Vs}$ and $\mathrm{\alpha }=11$ (black dashed line), within the range of $\mathrm{N}$ covered by the seed films. Therefore, we observe in Fig. 2 that single-pulse ReLA at $\le 75 \; {\mathrm{mJ cm}}^{-2}$ provides almost no alteration of the carrier transport properties from the seed ITO film. ReLA at $100 \; {\mathrm{mJcm}}^{-2}$ results in a minor improvement to the film quality (${\upmu }_{\mathrm{min}}$ is increased to $60 \; \mathrm{ c}{\mathrm{m}}^{2}/\mathrm{Vs}$; black arrow in Fig. 2). ReLA at $125 \; {\mathrm{mJ cm}}^{-2}$ appears to cross a critical fluence threshold which leads to an even greater improvement of the film quality (${\upmu }_{\mathrm{min}}$ is increased to $90 \; \mathrm{ c}{\mathrm{m}}^{2}/\mathrm{Vs}$) alongside an apparent reduction in the “strength” of ICS (${\alpha }$ is reduced from $11$ to $6$). These enhancements of the film quality likely arise from either structural and/or compositional modifications^16,17^, to be discussed later. Furthermore, for the films annealed at $125 \; {\mathrm{mJ cm}}^{-2}$, a change in the mobility alone (grey arrow) is not able to explain the post-ReLA transport properties. To do so, an additional change in ${\mathrm{N}}_{\mathrm{opt}}$ is required. Vitally, the increase in ${\mathrm{N}}_{\mathrm{opt}}$ in an oxidising atmosphere ($100\mathrm{\%}$ O_2_; green arrow) is less than in a reducing atmosphere ($5\mathrm{\%}$ H_2_ in N_2_; red arrow), indicating that this translation is ambient-dependent.

To assess the ambient-dependence further, we performed an additional single-pulse ReLA at $125 \; {\mathrm{mJ cm}}^{-2}$ in an intermediately oxidising environment ($5\mathrm{\%}$ O_2_ in N_2_). We present the results of this investigation with the blue square in Fig. 2. The post-ReLA transport characteristics for this sample are found to lie on the same cluster with $\mathrm{N}$ between that of the samples annealed at $125 \; {\mathrm{mJ cm}}^{-2}$ in oxidising and reducing environments (blue arrow), confirming that intermediate mixtures of reducing and oxidising gasses results in intermediate carrier concentrations. Further investigations into the precise relation between the oxygen partial pressure and additional ambient atmospheres (e.g., N_2_, Ar, Vacuum) are highly warranted, but are beyond the scope of this work. In summary, ReLA induces modifications of the carrier transport properties of the ITO film. This comprises an enhancement of ${\upmu }_{\mathrm{opt}}$ at $\ge 100 \; {\mathrm{mJ cm}}^{-2}$ coupled with an ambient-dependent modulation of $\mathrm{N}$ at $125 \; {\mathrm{mJ cm}}^{-2}$. The increase in $\mathrm{N}$ was greater for the environments with less oxygen. The modifications of the carrier transport properties translate into changes in the complex permittivity of ITO (see Supplementary Fig. S15), which has implications for the potential of ITO as a plasmonic material component^29,30^.

### Structural ReLA-induced modifications

**Figure 3:**
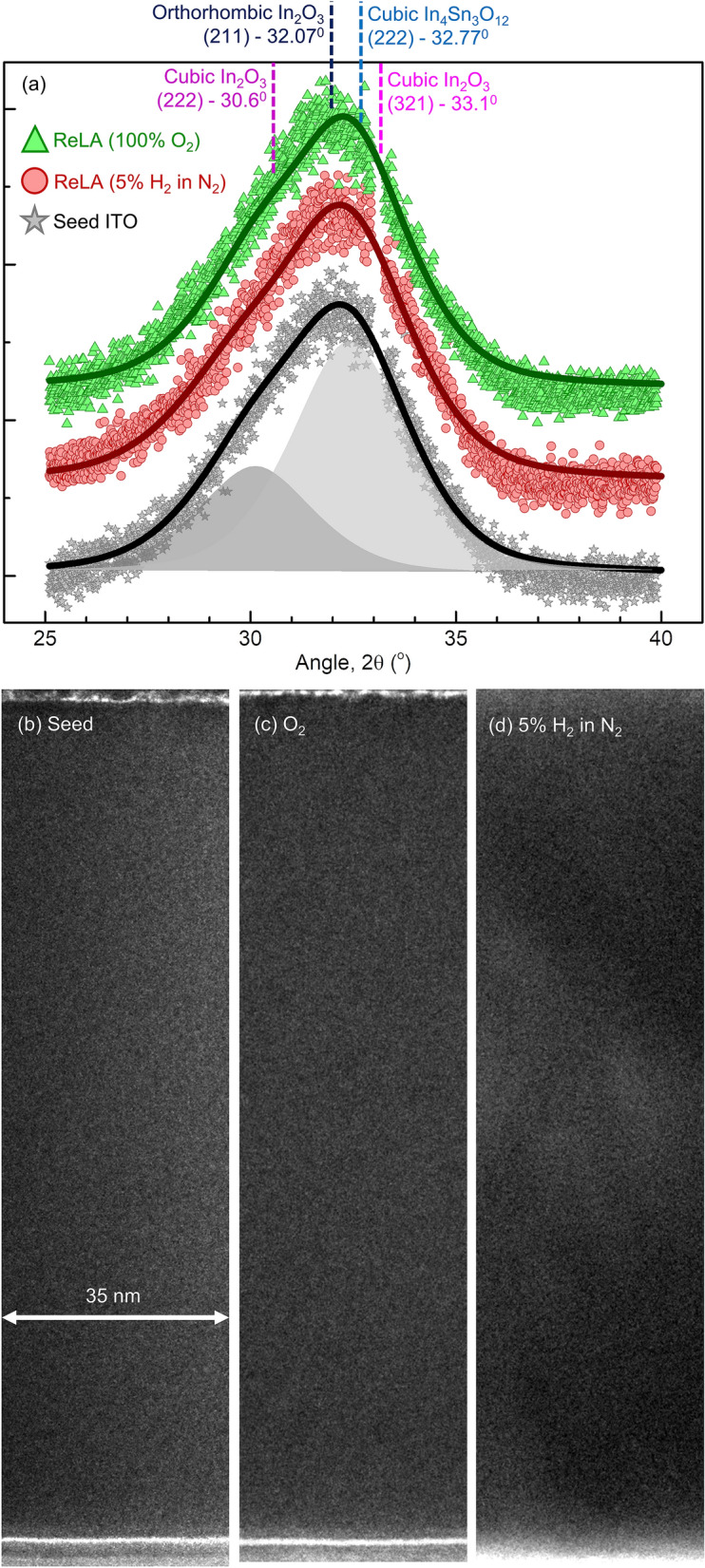
Structural properties of seed ITO films and laser processed at $125 \; \mathrm{ mJ c}{\mathrm{m}}^{-2}$. X-ray diffractograms for seed ITO film (grey stars) and those subject to ReLA in $5\mathrm{\%}$ H_2_ in N_2_ (red circles) and $100\mathrm{\%}$ O_2_ (green triangles) at $125 \; {\mathrm{mJ cm}}^{-2}$. The solid black, red, and green lines represent the corresponding fit of two pseudo-Voigt functions to the data. For the seed film, we present the component peaks, representing cubic In_2_O_3_ ($222$) and rhombohedral In_4_Sn_2_O_12_, with the grey shaded areas. The sharp ($200$) Si peak at $32.9^\circ {-}\:33.1^\circ$ has been manually removed from all diffractograms. Also shown are the TEM cross-sectional images of the (**b**) seed ITO film and those subject to ReLA at $125 \; {\mathrm{mJ cm}}^{-2}$ in (**c**) $100{\%}$ O_2_ and (**d**) $5{\%}$ H_2_ in N_2_.

To examine whether these modifications arise from changes in the crystal structure, we investigated the crystallinity of the seed and processed films with XRD. Figure 3a presents the X-ray diffractograms for the seed film (grey squares) and the corresponding films processed with a single laser pulse at $125 \; {\mathrm{mJ cm}}^{-2}$ in $100\%$ O_2_ (green squares) and in $5{\%}$ H_2_ in N_2_ (red squares). The X-ray diffractograms for all samples can be found in the Supplementary, section D.

The observed broad, asymmetric peak at $\sim 32.5^\circ$ may arise through various ways: (i) a mixing of ($222$) cubic In_2_O_3_ ($\mathrm{a}=1.0117 \; \mathrm{ nm}$^31^, $2\uptheta =30.61^\circ$) and ($321$) cubic In_2_O_3_ ($\mathrm{a}=1.0118 \; \mathrm{ nm}$^31^, $2\uptheta =33.127^\circ$; pink dashed line Fig. 3a) preferred orientations (ii) a mixing of ($222$) In_2_O_3_ and In_4_Sn_3_O_12_ ($\mathrm{a}=0.9467 \; \mathrm{ nm}$^32^, $2\uptheta =32.77^\circ$; blue dashed line) phases, present in amorphous ITO films^33–36^, and/or (iii) a ($211$) preferred orientation for orthorhombic In_2_O_3_ ($\mathrm{a}=0.7912 \; \mathrm{ nm}$, $\mathrm{b}=0.5477 \; \mathrm{ nm}$, $\mathrm{c}=0.5592 \; \mathrm{ nm}$^37^, $2\uptheta =32.07^\circ$; dark blue dashed line). To account for the asymmetry and potential mixing, the diffractograms are fit with two *pseudo*-Voigt functions (grey shaded areas in Fig. 3a) with shared Lorentzian and Gaussian broadening factors (${\upbeta }_{\mathrm{L}}$ and ${\upbeta }_{\mathrm{G}}$, respectively)^38^. Employing Scherrer’s formula^39^, we extract a grain size, $\mathrm{L}$, from the Lorentzian integral breadth, ${\uptau }_{\mathrm{L}}=(\uppi /2){\upbeta }_{\mathrm{L}}$, that has been reported to give more accurate determination of $\mathrm{L}$^38^. For the seed ITO film, we find that $\mathrm{L}=3.2\pm 0.3 \; \mathrm{ nm}$, confirming that the film is nanocrystalline. The offset of the ($222$) peak from the “bulk” value (dark pink dashed line in Fig. 3a) indicates that the film is under tensile stress (${\updelta }_{\mathrm{S}}=7.0\pm 0.3 \; \mathrm{GPa}$) due to the interfacial mismatch between the atomic spacings for the Si and In_2_O_3_ lattices^40^. From this, we calculate the “bulk” peak position for the 2nd, larger, peak to be $33.17^\circ \pm 0.04^\circ$, indicating that the 2nd peak likely arises from a ($321$) preferred orientation for cubic In_2_O_3_. However, we cannot exclude the possibility of a co-existence of all three potential sources mentioned above. To summarise, the observed large peak broadening and asymmetry for the seed film arises from the nanocrystalline nature of the room temperature deposited ITO films film. In this case, we note from Fig. 3a that the nanocrystalline nature is unaffected during ReLA at $\le 125 \; \mathrm{ mJ c}{\mathrm{m}}^{-2}$ in either atmosphere. Indeed, there is no resolvable change in the internal stress, grain size, or the ratio of the two component peak areas for any of the laser processed ITO films (Supplementary, section D).

However, XRD averages over the entire sample and, due to the inherent depth-dependence of the LA process (see Supplementary, section F), the modifications may be highly localised. To elucidate any microscopic modifications to the film structure, we examined high-resolution TEM images for the seed ITO thin film (Fig. 3b) and the ITO films subjected to single-pulse processing at $125 \; {\mathrm{mJ cm}}^{-2}$ in $100\mathrm{\%}$ O_2_ (Fig. 3c) and $5\%$ H_2_ in N_2_ (Fig. 3d) environments. All the samples are confirmed to be nanocrystalline and appear uniform across the entire sample depth, both before and after ReLA at $125 \; {\mathrm{mJ cm}}^{-2}$. It should be noted that in previous reports on LA of ITO, structural changes have been observed at lower fluences^16,17,41^. The deviation between these reports and this work could may be due to the different thickness of the ITO film and the intermediate SiO_2_ layer (acting as a thermal barrier and an optical spacer), and/or the growth process (solution versus sputtering)^16,17,41^. However, we also note that an increased ambient pressure is typically employed to suppress and/or eliminate sample ablation during LA^42,43^. We conclude that ReLA, at high pressure and up to and including $125 \; {\mathrm{mJ cm}}^{-2}$ in either $5{\%}$ H_2_ in N_2_ or $100{\%}$ O_2_, is a “low-stress” process and the modifications to the optoelectronic properties induced during ReLA cannot be explained through structural changes.

### Compositional ReLA-induced modifications

As the structure of the ITO films does not significantly change during ReLA, we focus our attention to the compositional alterations. To elucidate the compositional changes that govern the tailoring of the optical properties of ITO, we examined the surface oxidation states of the individual elements, via XPS, for the seed ITO film and one that was subjected to single-pulse ReLA at $125 \; {\mathrm{mJ cm}}^{-2}$ in the reducing environment ($5{\%}$ H_2_ in N_2_). A key dominant donor mechanism in ITO is the introduction of the Sn dopant to In_2_O_3_^20^. Therefore, we initially examine the peak in the core-level X-ray photoelectron spectra (see Supplementary, section E) that is related to Sn atoms. We deconvolute the Sn3d_5/2_ peak and extract the areal intensities of the Sn^4+^ and Sn^2+^ components, which are related to the “activated” SnO_2_ and the “un-activated” Sn–O within the film, respectively^7^. As the donor state of ITO originates from Sn^4+^ ions substituting into In^3+^ and oxygen vacancy positions (becoming “activated”), the areal intensity of the Sn^4+^ component can be related to adjustments of the carrier concentration^44,45^.

**Figure 4:**
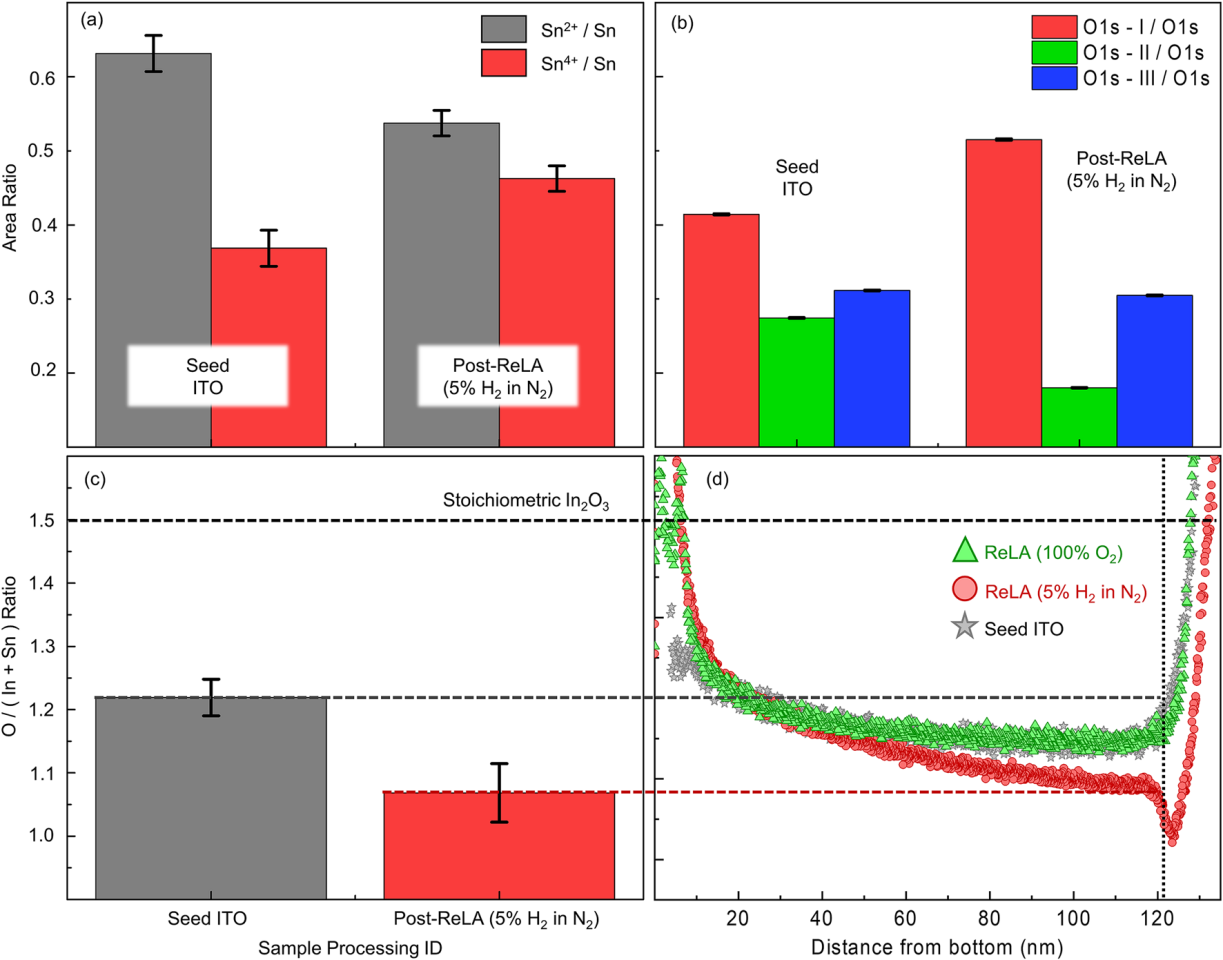
Compositional properties of seed ITO films and laser processed at $125 \; \mathrm{ mJ c}{\mathrm{m}}^{-2}$. (**a,b**) Relative areal intensities of the deconvoluted component peaks of the core level X-ray photoelectron spectra for Sn:Sn^2+^ (grey bar) and Sn^4+^ (red bar) and O1s peaks: O_I_ (red bar), O_II_ (green bar) and O_III_ (blue bar), respectively, for the seed ITO film and that subject to a single ReLA pulse at $125 \; {\mathrm{mJ cm}}^{-2}$ in $5{\%}$ H_2_ in N_2_. (**c**) The $\mathrm{O}/(\mathrm{In}+\mathrm{Sn})$ ratio for the seed ITO film (grey bar) and that subject to a single ReLA pulse at $125 \; {\mathrm{mJ cm}}^{-2}$ in $5{\%}$ H_2_ in N_2_ (red bar), calculated from surface-XPS results. (**d**) Depth profile of $\mathrm{O}/(\mathrm{In}+\mathrm{Sn})$ for the seed ITO film (grey stars) and those subject to single ReLA pulse at $125 \; {\mathrm{mJ cm}}^{-2} \;$ in $5{\%}$ H_2_ in N_2_ (red circles) and $100{\%}$ O_2_ (green triangles), as calculated from EDX imaging. $\mathrm{O}/(\mathrm{In}+\mathrm{Sn})$ for a stoichiometric In_2_O_3_ film is indicated by the black dashed line. We map the XPS results onto the “surface” (black vertical dotted line) of the EDX depth profile with the horizontal grey and red dashed lines.

We present the areal intensities of the Sn^4+^ and Sn^2+^ components in relation to the Sn3d_5/2_ peak in Fig. 4a**.** We note that in the seed film the Sn^2+^ component is dominant and that there is an increased Sn^4+^ content after ReLA in $5{\%}$ H_2_ in N_2_. Evidently, Sn activation at $125 \; \mathrm{ mJ c}{\mathrm{m}}^{-2}$ accounts for the enhancement of ${\mathrm{N}}_{\mathrm{opt}}$ observed in Fig. 2. We infer that a similar process of Sn activation occurs for the film processed in $100{\%}$ O_2_, resulting in the increase in ${\mathrm{N}}_{\mathrm{opt}}$ (Fig. 2). This is despite the highly oxidising environment, which can be reasonably expected to reduce ${\mathrm{N}}_{\mathrm{opt}}$ by filling oxygen vacancies^10–14^. To investigate the role of the oxygen vacancies as donor states, we examine the O1s core-level X-ray photoelectron peak (see Supplementary, section E, for the measurements and peak fits). The O1s peak is de-convoluted into O_I_, O_II_ and O_III_ components^7^. The O_I_ and O_III_ components are related to the metal-oxide binding (In-O)^45^ and surface contaminants^46^, respectively^41^. The origin of the O_II_ peak is commonly associated with oxygen atoms existing near to a neighbouring oxygen vacancy. However, this is still under debate, and it is also possible that the O_II_ peak is assigned to the amorphous phase of ITO and/or oxygen atoms bound to Sn^10,47,48^. The relative areal intensities of the O1s components are shown in Fig. 4b. The film subjected to ReLA in $5{\%}$ H_2_ in N_2_ exhibits an enhancement of the metal oxide peak (O_I_; red bar in Fig. 4b) in relation to the seed film. This corresponds to a removal of oxygen interstitials that activates the Sn^4+^ donors and corroborates the improvement in ${\upmu }_{\mathrm{opt}}$ observed in Fig. 2 (particularly the increased ${\upmu }_{\mathrm{min}}$)^49^. There is also a marked decrease in the relative areal intensity for the peak commonly associated with oxygen vacancies (O_II_, green bar in Fig. 4b). As the decrease in the O_II_ peak corresponds to a reduction in the amount of oxygen (relative to In and Sn) alongside an increase in the carrier concentration, it seems unlikely that the O_II_ peak is a result of oxygen vacancies. In addition, the lack of a significant change in the structure (Fig. 3) eliminates the amorphous phase as the source of the O_II_ peak. The correlation between the reductions in the relative areal intensity of the O_II_ peak and the increase in the Sn^4+^/Sn^2+^ ratio suggests, instead, that the oxygen atoms bound to Sn are the primary source. It is, however, possible that the peak arises from a summation of the above-mentioned sources, which cannot be individually resolved during the peak fitting. Finally, there exists a reduction in the surface contaminants after annealing in $5{\%}$ H_2_ in N_2_ (O_III_; blue bar in Fig. 4b). Due to the surface nature of XPS, it is somewhat unclear how these modifications correlate to the bulk of the ITO film.

To reveal the compositional modifications induced across the entire depth of the sample we turn to the EDX measurements, presented in Fig. 3. Across the image, the average emitted X-ray intensity at the characteristic energy of each element within the sample was measured in order to build a depth profile of the relative elemental concentration across the film. The intensities, plotted relative to their maxima, are presented in the Supplementary, section E. To calibrate the EDX intensities to the absolute surface region abundance determined via XPS, we first calculate the surface region $\mathrm{O}/(\mathrm{In}+\mathrm{Sn})$ ratio from the total areal intensities of the O1s, In3d_3/2_, In3d_5/2_ Sn3d_3/2_, and Sn3d_5/2_ XPS peaks (considering the relative sensitivity factors)^50,51^. We present the surface region $\mathrm{O}/(\mathrm{In}+\mathrm{Sn})$ ratio for the seed ITO film (grey bar) and that subject to single-pulse ReLA at $125 \; {\mathrm{mJ cm}}^{-2}$ in $5{\%}$ H_2_ in N_2_ (red bar) in Fig. 4c. The likelihood that annealing in $5{\%}$ H_2_ in N_2_ has created new oxygen vacancies is supported by the drop in the relative O content, from 1.21 to 1.06. Both values are a departure from the stoichiometric case of $\mathrm{O}/(\mathrm{In}+\mathrm{Sn})=1.5$ (dashed black line in Fig. 4c). Significantly, the observed modulation of the oxygen vacancy contribution may give rise to the ambient dependence of the change in the carrier concentration during ReLA. However, this is only relevant to the surface of the film and the ReLA process is depth-dependent (see Supplementary, section F). Figure 4c is used to calibrate the EDX-calculated depth profile of the normalised $\mathrm{O}/(\mathrm{In}+\mathrm{Sn})$ ratio across the sample depth for the seed ITO film (grey stars) and those subject to single-pulse ReLA at $125 \; {\mathrm{mJ cm}}^{-2}$ in $5{\%}$ H_2_ in N_2_ (red circles) and in $100{\%}$ O_2_ (green triangles).

We present this as a function of the distance from the bottom of the film in Fig. 4d. We offset the EDX results so that the values of $\mathrm{O}/(\mathrm{In}+\mathrm{Sn})$ for the seed and $5{\%}$ H_2_ in N_2_ annealed film, at roughly $10 \; \mathrm{ nm}$ below the imaged surface (to account for the sampling depth)^52^, match the values determined from XPS. Further details on the analysis of the EDX images is presented in the Supplementary, section E. These steps allow us to examine how the oxygen content varies across the film. In Fig. 4d, a smooth gradient is observed in the relative oxygen concentration across the seed film. The gradient in $\mathrm{O}/(\mathrm{In}+\mathrm{Sn})$ is more substantial for the film processed in $5{\%}$ H_2_ in N_2_, and that near the surface there is a sharp dip in $\mathrm{O}/(\mathrm{In}+\mathrm{Sn})$. Such a gradient has been reported previously using secondary ion mass spectroscopy^53^, where a dip of $\mathrm{O}/(\mathrm{In}+\mathrm{Sn})$ at the surface of the film was also found for reducing annealing environments. The gradient of the oxygen throughout the depth of the film is reflected in the presence of the simply graded inhomogeneity utilised during SE analysis.

### Reflecting the oxygen gradient in the ellipsometric model

**Figure 5:**
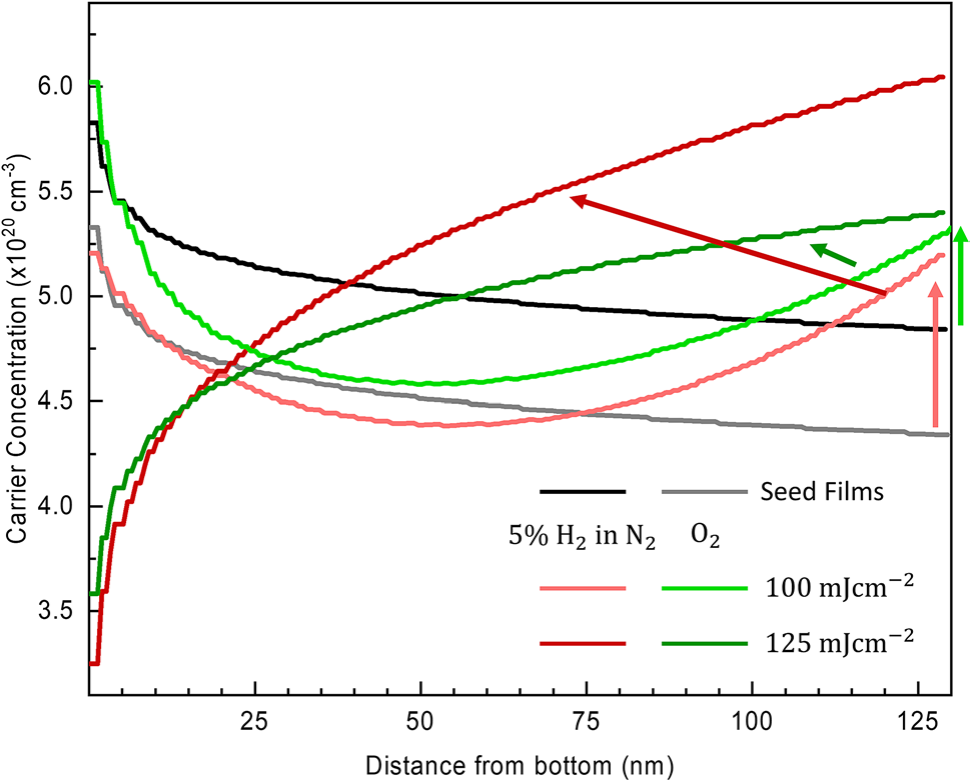
The optical carrier concentration as a function of the distance from the ITO/Si interface, ${\mathrm{N}}_{\mathrm{opt}}(\mathrm{z})$, for two representative samples at different positions on the seed ITO film (grey and black line) and those subject to single-pulse ReLA at $100 \; {\mathrm{mJ cm}}^{-2}$ (lighter shade) and $125 \; {\mathrm{mJ cm}}^{-2}$ (darker shade) in both $5{\%}$ H_2_ in N_2_ (red line) and $100{\%}$ O_2_ (green line).

To reflect the observed gradient in the oxygen content across the film (Fig. 4d), an exponential gradient in ${\uprho }_{\mathrm{opt}}$ and ${\uptau }_{\mathrm{opt}}$ across the depth of the ITO films was included in the ellipsometric analysis using Eq. (4) and (5).

4 $${\uprho }_{\mathrm{opt}}\left(\mathrm{z}\right)={\uprho }_{\mathrm{opt},0}\left(1+\frac{{\uprho }_{\mathrm{\%Gr}}}{100}{\left(\frac{\mathrm{z}}{{\mathrm{d}}_{\mathrm{B}}}\right)}^{\upchi }\right)$$

5 $${\uptau }_{\mathrm{opt}}\left(\mathrm{z}\right)={\uptau }_{\mathrm{opt},0}\left(1+\frac{{\uptau }_{\mathrm{\%Gr}}}{100}{\left(\frac{\mathrm{z}}{{\mathrm{d}}_{\mathrm{B}}}\right)}^{\upchi }\right)$$

where ${\uprho }_{\mathrm{opt},0}$ and ${\uptau }_{\mathrm{opt},0}$ are the values of the optical resistivity and mean free time, respectively, at the bottom of the film (the ITO/$\mathrm{Si}$ interface). ${\uprho }_{\mathrm{\%Gr}}$ and ${\uptau }_{\mathrm{\%Gr}}$ are the percent gradient of the optical resistivity and mean free time, respectively. $\mathrm{z}$ is the distance from the bottom of the layer, ${\mathrm{d}}_{\mathrm{B}}$ is the layer thickness, and $\upchi$ is the exponent of the gradient. The gradient is described by a series of $100$ discrete slices such that $\mathrm{z}=\left(\mathrm{c}+0.5\right)/\mathrm{n}$ where $\mathrm{c}$ is the current slice, $\mathrm{n}$ is the total number of slices and $\mathrm{z}$ falls at the middle of each slice. The resulting MSE for fitting this film using a gradient in ${\uprho }_{\mathrm{opt}}$ and ${\uptau }_{\mathrm{opt}}$ was reduced (from $14.7$ to $10.3$). The values of ${\mathrm{N}}_{\mathrm{opt}}(\mathrm{z})$ were calculated from ${\uprho }_{\mathrm{opt}}(\mathrm{z})$ and ${\uptau }_{\mathrm{opt}}(\mathrm{z})$ and are presented in Fig. 5 for the two of the seed optimised ITO films (black and grey line) and those subject to ReLA in $5{\%}$ H_2_ in N_2_ (red line) and $100{\%}$ O_2_ (green line) at $100 \; \mathrm{ mJ c}{\mathrm{m}}^{-2}$ (lighter shade) and $125 \; \mathrm{ mJ c}{\mathrm{m}}^{-2}$ (darker shade). The films annealed at $<100 \; \mathrm{ mJ c}{\mathrm{m}}^{-2}$ showed a negligible change in the gradient of ${\mathrm{N}}_{\mathrm{opt}}(\mathrm{z})$ as compared to the seed films, and so are excluded from Fig. 5 for the sake of clarity. For the seed ITO films (black and grey solid lines in Fig. 5), ${\mathrm{N}}_{\mathrm{opt}}(\mathrm{z})$ is larger towards the bottom of the film where it rapidly falls by $\sim 1\times {10}^{20} \; \mathrm{ c}{\mathrm{m}}^{-3}$ within the first $\sim 10 \; \mathrm{nm}$ before coming to a low plateau. A sharper gradient in ${\mathrm{N}}_{\mathrm{opt}}(\mathrm{z})$ near the bottom of the film is observed for the film annealed at $100 \; \mathrm{ mJ c}{\mathrm{m}}^{-2}$ in both the reducing and oxidising environments (light shaded red and green, respectively, lines in Fig. 5). At the surface of the film, however, ${\mathrm{N}}_{\mathrm{opt}}$ is increased from the case of the seed films, and this results in an almost parabolic shape of ${\mathrm{N}}_{\mathrm{opt}}(\mathrm{z})$ across the film, where there is a lower ${\mathrm{N}}_{\mathrm{opt}}$ near the middle of the film.

The local minimum in ${\mathrm{N}}_{\mathrm{opt}}(\mathrm{z})$ arises from the interaction between a decreasing ${\uprho }_{\mathrm{opt}}(\mathrm{z})$ and ${\uptau }_{\mathrm{opt}}(\mathrm{z})$. As was seen in Fig. 2, the “average” ${\mathrm{N}}_{\mathrm{opt}}$ of the film annealed at $100 \; \mathrm{ mJ c}{\mathrm{m}}^{-2}$ is larger for the film annealed in $100\%$ O_2_ rather than $5{\%}$ H_2_ in N_2_. However, a reasonable assumption can be that the films used for laser processing for each ambient were slightly different due to the slight disuniformity of the deposition. This can be seen to occur by noting that ${\mathrm{N}}_{\mathrm{opt}}$ at the bottom of the film is larger for the film annealed in $100\%$ O_2_, and how it is very similar to the seed film with the largest ${\mathrm{N}}_{\mathrm{opt}}$ (black line). For ReLA in $5{\%}$ H_2_ in N_2_, ${\mathrm{N}}_{\mathrm{opt}}(\mathrm{z}=0)$ conversely is very similar to the seed film with the smallest ${\mathrm{N}}_{\mathrm{opt}}$ (black line). By accounting for this, it is seen that the increase of ${\mathrm{N}}_{\mathrm{opt}}$ at the surface (relative to the seed film) is larger for the film annealed in $5{\%}$ H_2_ in N_2_, than for the film annealed in $100{\%}$ O_2_. This is indicated by the light shaded red and green arrows in Fig. 5. For the films annealed at $125 \; \mathrm{ mJc}{\mathrm{m}}^{-2}$ in $5{\%}$ H_2_ in N_2_ and in $100{\%}$ O_2_ (dark red and green lines in Fig. 5, respectively), ${\mathrm{N}}_{\mathrm{opt}}$ at the surface is increased even further and the increase is, again, dependent on the ambient. The steepness of the ${\mathrm{N}}_{\mathrm{opt}}(\mathrm{z})$ curve for the seed and laser processed films agree well with the trend in the $\mathrm{O}/(\mathrm{In}+\mathrm{Sn})$ ratio observed in Fig. 4d. This is expected from the increased likelihood of oxygen vacancy donors in the oxygen deficient lattice. Closer to the substrate, ${\mathrm{N}}_{\mathrm{opt}}(\mathrm{z})$ is reduced in comparison to the seed film for both the films annealed in $5{\%}$ H_2_ in N_2_ and $100\%$ O_2_. This is reflected by how the $\mathrm{O}/(\mathrm{In}+\mathrm{Sn})$ ratio is increased at the bottom of the film (Fig. 4d). The overall trend can be described by a shift of the gradient to lower in the film, so that the “dip” in ${\mathrm{N}}_{\mathrm{opt}}$ lies near the bottom of the film.

These results are used to build a physical interpretation of the ReLA process. In response to the photo-induced localised heating, the temperature at the surface rises sufficiently (see Supplementary, section F) to allow the oxygen atoms to become mobile within the lattice. In the reducing environment, the oxygen overflows from the thin films into the environment. This causes the reduced oxygen content in the surface region, resulting in the formation of oxygen vacancies^54^. While the temperature within the film is high enough, oxygen atoms remain mobile and they continue to be drawn towards this region, resulting in the final increased oxygen gradient within the film. In the oxidising environment, however, the oxygen atoms that escape at the surface are quickly replaced with oxygen atoms from within the pressurised chamber. Therefore, the system (regarding the distribution of oxygen atoms) is at equilibrium and the oxygen content across the film remains highly similar to that of the seed film. Depth-profile EDX does not elucidate whether the oxygen atoms within the film are more likely to end up in metal oxide or interstitial positions after ReLA. However, from the increased mobility observed in the optical results (Fig. 2) we can infer a reduction in oxygen interstitial concentration as no structural modifications were observed (Fig. 3). Finally, it is important to note that annealing in hydrogen has a potential to promote hydrogen doping within the lattice^54,55^. This has been proposed to be a shallow donor that is more energetically favourable than oxygen vacancies^56^. The effect of H-doping during ReLA in $5{\%}$ H_2_ in N_2_ may further enhance the carrier concentration modulation, but analysis of this mechanism is beyond the scope of this work. From this physical picture, we conclude that the ambient dependence of the free carrier modulation during ReLA arises primarily from the probing of the oxygen vacancies. In reducing environments, the oxygen that becomes mobile in the film escapes, leaving behind increased oxygen vacancies, increasing ${\mathrm{N}}_{\mathrm{opt}}$. However, due to the activation of Sn^4+^ within the film, we note an increase in the carrier concentration even within oxidising environments. As single-pulse ReLA in $100\%$ O_2_ does not change the oxygen content within the film from that of the seed film, we are unable here to reduce the carrier concentration. For applications where a lower carrier concentration is desired in order to shift the plasma energy to lower photon energy, such as for low-loss NIR and IR plasmonics^29,30,57^, a different strategy is required.

## Conclusions

It was shown that ReLA offers a low stress method to engineer the defects of the ITO films by enhancing $\upmu$ and selectively increasing $\mathrm{N}$. The modifications arise from a combination of Sn^4+^ activation and manipulation of the oxygen migration during the annealing process. The low-stress nature of the process means that ReLA can promote defect mobility within the lattice without causing structural changes. This potentially makes the technique applicable to more complex prefabricated nano/microstructures, where changes to the structure may degrade device performance. Furthermore, the low-thermal budget of LA allows for the application of ReLA to annealing of materials on heat sensitive substrates, such as those used for flexible electronics, provided that the bulk of the thermal dose is contained within the ITO film (Supplementary, section F)^58,59^. It should be noted that this is just for a single pulse and as such, multi-pulse ReLA is likely to be able to enhance the capability to engineer the defects of TCOs and thus tailor their carrier transport properties. Further investigations into the precise role of an intermediate oxygen partial pressure during ReLA and/or other ambient environments are also warranted.

## Methods

### Thin film deposition

ITO films (thickness of $\sim 130 \; \mathrm{ nm}$) were deposited onto double side polished n-type ($1{-}10 \; { \Omega {\rm cm}}$), ${4}^{{{\prime \prime}}}$, ($100$) oriented Si wafers with a $2 \; \mathrm{ nm}$ native oxide using radio frequency magnetron sputtering. The base pressure was $\sim {10}^{-5} \; \mathrm{ Pa}$ ($\sim {10}^{-7} \; \mathrm{ mbar}$). Intentional substrate heating or bias was not applied to the substrate. We utilised a ${3}^{{\prime \prime}}$ target of $10 \; \mathrm{ wt}.{\%}$ Sn:In_2_O_3_ ($99.99{\%}$ purity). Ar and O_2_ were introduced to the chamber with an O_2_ concentration of $0.25\pm 0.02{ \%}$ and the deposition was performed with a sputtering pressure of $2 \; \mathrm{ mTorr}$ and an RF power of $40 \; \mathrm{ W}$. The substrate to target distance was $10.8\pm 0.2 \; \mathrm{ cm}$. Further details on the deposition process can be found elsewhere^60,61^. The seed film exhibited non-uniformity in the thickness and resistivity across the Si wafer (see Supplementary, section A). To account for this, the wafer was diced into $\sim 8\times 8 \; \mathrm{mm}^{2}$ die and a cluster of the seed films with the most similar resistivity were selected for further processing. Two samples with resistivities at the extreme ends of this cluster ($4.89-5.4\times {10}^{-4} \; { \Omega {\rm cm}}$) remained un-processed to represent the seed film. These samples are denoted as “S1” and “S2” for the higher and lower resistivity seed films, respectively, within the manuscript and the supplementary.

### Reactive laser annealing

LA was performed at room temperature with a KrF ($248 \; \mathrm{ nm}$) excimer laser (LAMBDA PHYSIK LPX 305i), which can deliver unpolarised light, with an average energy per pulse of up to $1.5 \; \mathrm{ J}$, at a $1{-}50 \; \mathrm{ Hz}$ pulse repetition rate and $25 \; \mathrm{ ns}$ pulse length (i.e., average power per pulse of $60 \; \mathrm{ MW}$) to the sample surface. Further details of the LA system are published elsewhere^60,61^. Here, we also utilise a pressure cell with a UV-transparent window to enable LA within pressurised ($6.89\times {10}^{5} \; \mathrm{ Pa}$ or $100 \; \mathrm{ psig}$) environments of $5\%$ H_2_ in N_2_ and $100{\%}$ O_2_. We call the process of LA within a reactive gas environment “reactive LA” (ReLA). For each ambient composition, a single laser pulse was delivered to the sample with a fluence, ${\mathrm{J}}_{\mathrm{L}}$, of $25{-}125 \; \mathrm{ mJ c}{\mathrm{m}}^{-2}$ (power density of $1{-}5 \; \mathrm{MW c}{\mathrm{m}}^{-2})$ in steps of $25 \; \mathrm{ mJ c}{\mathrm{m}}^{-2}$. We also performed single-pulse ReLA at $125 \; {\mathrm{mJ cm}}^{-2} \;$ in $5{\%}$ O_2_ in N_2_. We note that $125 \; \mathrm{ mJ c}{\mathrm{m}}^{-2}$ was the maximum fluence allowed by our experimental setup that could maintain a readily measurable spot size of $1.20\times 1.20 \; {\mathrm{cm}}^{2}$.

### Thin film characterisation

To reveal a complete picture of the laser-matter interactions that govern the conversion of the optoelectronic properties of the seed samples, an extensive set of characterisation techniques were utilised to relate the optical properties to the electronic, structural, compositional, and morphological modifications of ITO during ReLA.

### Optoelectronic characterisation

Optical characterisation was performed using a J. A. Woollam Mark II infrared (IR)-SE in the spectral range of $0.034{-}0.8 \; \mathrm{ eV}$ ($1.4{-}40 \; { \upmu {\rm m}}$) and a J. A. Woollam M2000 SE in the spectral range of $0.74{-}3.34 \; \mathrm{ eV}$ ($0.37{-}1.7 \; { \upmu {\rm m}}$) at incident angles of $65^\circ$, $70^\circ$, and $75^\circ$. An optical model, reflecting the sample geometry, comprised the complex permittivity, $\widetilde{\upvarepsilon }(\mathrm{E})$, of each material and was fitted to $\Psi (\mathrm{E})$ and $\Delta (\mathrm{E})$. From the fitting process, we extract the geometric features (film thickness, surface roughness and uniformity) and the parameterised oscillators that describe $\widetilde{\upvarepsilon }(\mathrm{E})$ (interband transitions, phonon modes and/or defect states absorption, free carriers etc.) for the ITO thin films^62^. The J. A. Woollam Mark II IR-SE and a normal-incidence optical reflectance probe were also utilised to measure the IR transmittance, ${\mathrm{T}}_{\mathrm{IR}}(\mathrm{E})$*,* and visible reflectance, ${\mathrm{R}}_{\mathrm{VIS}}(\mathrm{E})$, respectively. Electrical characterisation of the resistivity was performed via 4pp in both collinear and Van-der-Pauw configurations (${\uprho }_{4\mathrm{pp}}$ and ${\uprho }_{\mathrm{Hall}}$, respectively). Characterisation of the “Hall” carrier concentration, ${\mathrm{N}}_{\mathrm{Hall}}$, was performed with an Ecopia HMS-3000 Hall Measurement System at room temperature ($\mathrm{B }= 0.553 \; \mathrm{ T}$). Further details of these techniques were provided in a recent publication^26^. It should be noted that the typical back-surface finish of single-side polished Si wafers is insufficiently rough to ensure that there are no substrate back-reflections during IR-SE measurements. To account for this, the backsides of the Si wafers were roughened using a Dremel tool with a diamond head (following measurements of ${\mathrm{T}}_{\mathrm{IR}}(\mathrm{E})$).

### Structural characterisation

Structural characterisation was performed by XRD employing a PanAnalytical Pro Diffractometer. The spot size was set to $5 \times 5 \; {\mathrm{mm}}^{2}$ via a $10 \; \mathrm{ mm}$ height-limiting slit, ${2}^{\mathrm{rad}}$ Soller slits and a programmable divergence slit. The scan step size was $0.0083556^\circ$ with a time per step of $1998.345 \; \mathrm{ s}$ in a range of $25^\circ {-} 40^\circ$. The very long step time was required due to the small sample size and amorphous nature of the room-temperature sputtered ITO films^9,53^. The range was chosen to cover the most clearly identifiable ($222$) and ($400$) Bragg peaks for bixbyite In_2_O_3_ ($\mathrm{a}=1.0118 \; \mathrm{ nm}$)^31^, at $30.6^\circ$ and $35.5^\circ$, respectively. Cross-sectional images of an indicative set of ITO thin films were obtained via high resolution TEM (FEI™ Talos sTEM) using a Schottky-type field emission gun operated with an electrostatic potential of $200 \; \mathrm{ kV}$. Prior to TEM, the films were subjected to focused ion beam milling process using a JEOL 4500 FIB/SEM.

### Compositional characterisation

Surface elemental characterisation was performed with a Kratos Analytical Ltd. AXIS Ultra XPS system using a monochromated Al-K_α1_ X-ray beam excitation source ($1486.6 \; \mathrm{ eV}$) in an ultra-high vacuum chamber (base pressure $\sim {10}^{-9} \; \mathrm{ bar}$). The relative elemental abundance across the entire depth of the ITO thin films was determined from EDX measurements with the FEI™ Talos sTEM.

## Supplementary Information

Supplementary Information.

## Data Availability

All data generated or analysed during this study are included in this published article and its supplementary information files.
