# Supplementary material for: Photo-engineered optoelectronic properties of indium tin oxide via reactive laser annealing
Source: Sci Rep. 2022 Sep 2;12:14986. doi: 10.1038/s41598-022-18883-5 (PMC9440247; doi:10.1038/s41598-022-18883-5)
Supplement: Supplementary file 1 — Supplementary Information. [file 41598_2022_18883_MOESM1_ESM.pdf]

Photo-engineered optoelectronic properties of indium tin oxide via reactive laser annealing

James A. Hillier[1], Panos Patsalas[2], Dimitrios Karfaridis[2], Sophie Camelio[3], Wayne Cranton[4], Alexei V. Nabok[4], Christopher J. Mellor[5], Demosthenes C. Koutsogeorgis[1], and Nikolaos Kalfagiannis*[1]

[1] Nottingham Trent University, School of Science and Technology, Nottingham, NG11 8NS, UK.
[2] Aristotle University of Thessaloniki, Department of Physics, Thessaloniki, GR-54124, Greece.
[3] Institut Pprime, UPR 3346 CNRS-Université de Poitiers, Poitiers, France.
[4] Sheffield Hallam University, Materials and Engineering Research Institute, Sheffield, S1 1WB, UK.
[5] The University of Nottingham, School of Physics and Astronomy, Nottingham, NG7 2RD, UK

* *nikolaos.kalfagiannis@ntu.ac.uk*

Contents

Section A: Thin Film Uniformity 2

Section B: Optical data 3

Parameterised Results 11

Section C: Ellipsometric modelling 14

Oscillator Functions 14

Justification of SE model 14

Section D: XRD data and analysis 16

XRD spectra for all ITO films 16

XRD analysis 17

Section E: XPS and EDX data and Analysis 18

XPS Data and fits 18

EDX data and analysis 18

References 20

Section A: Thin Film Uniformity

For the seed ITO films, some slight non-uniformity in the thickness and ρ_{4pp} was present across the 4″ wafers. This non-uniformity introduces a problem for further LA experiments which require a set of laser-processed "spots" of $\sim 1 \times 1$ cm across the full wafer. For example, deviations in the seed film may be mistaken for laser-induced modifications from the seed film to the annealed film. To account for this, we investigated into the non-uniformity of the film. After deposition of the ITO films, the wafers were cut into ~90, $\sim(8 \times 8)$ mm^2 die. The die were cut along their long edge for each cut, into to reduce any stresses introduce to the film. The x and y position, relative to the sample centre, was noted and tracked for each die. Following this, we measured the visible reflectance, R_{Vis}, IR transmittance, T_{IR}, for each die in order to determine the film thickness, d_{film}, "optical" resistivity, ρ_{opt}, "optical" mean free time, τ_{opt},and the optical constants, ε_1 and ε_2. The thickness, as determined from fitting of R_{Vis}, was used to calculate the collinear resistivity, ρ_{4pp}, from four-point probe (4pp) measurements of the sheet resistance. Furthermore, d_{film}, the UV oscillators, and the high-frequency permittivity, ε_∞, were fixed during the fitting of IRT data. The properties of each die were plotted against their x and y position to produce a set of colour maps. The deviation is calculated as the percent difference of the die to the average value. We calculate of the "optical" carrier concentration, N_{opt}, and mobility, μ_{opt}, via Eq. 2 and Eq. 3, respectively, from which an understanding of the variations in the transport mechanisms (that are more fundamental properties than the resistivity) can be built. The absolute value of the effective mass (assumed to be to be 0.30 m_e)[1] does not affect the resulting % deviation, but it is important to note that variations in N_{opt} and μ_{opt} may partially originate from variations in m_e^*. Figure S1a-d presents a colour map of the deviation in (a) d_{film}, (b) ρ_{opt}, (c) N_{opt}, and (d) μ_{opt}, across the 4″ wafer. In Figure S1a, we observe that the thickness range does not deviate more than 8 nm (7.2%) across the wafer and the standard deviation is only 2 nm (1.8%). It is well known that there exist a thickness dependence on the resistivity of ITO films[2] Furthermore, the change in thickness across the wafer implies a change in the sputtering rate, which has been noted to drastically affect the resulting film properties.[3,4] In Figure S1b, we observe a minor deviation in ρ_{4pp} across the wafer, where the average resistivity is lower towards the center. From Figure S1c-d, it is found that the decrease in ρ_{opt} towards the centre of the wafer arises primarily from an increase in μ_{opt} from ~15 cm^2/Vs to nearly twice the edge value of ~25 cm^2/Vs at the centre. The carrier concentration remains near constant with a value of ~4.2 cm^{-3}. It is apparent that the change in μ_{opt} gives rise to the decreasing resistivity with sputtering rate. As the film is amorphous across the entire wafer (Section D), structural alterations cannot be used to explain the change in μ_{opt} across the film. Instead, it is noted that for ITO films with $N > \sim 4 \times 10^{20}$cm^{-3}, the dominant scattering mechanism is ionized cluster scattering (ICS) that has a very large scattering coefficient for ITO films and thus the mobility is highly sensitive to N.[5,6] Because of this observed disuniformity across the wafer, the die selected for further processing were limited to an area of the film where the deviation was minimal. In this area, two die were selected to cover the range of resistivity exhibited by this cluster.

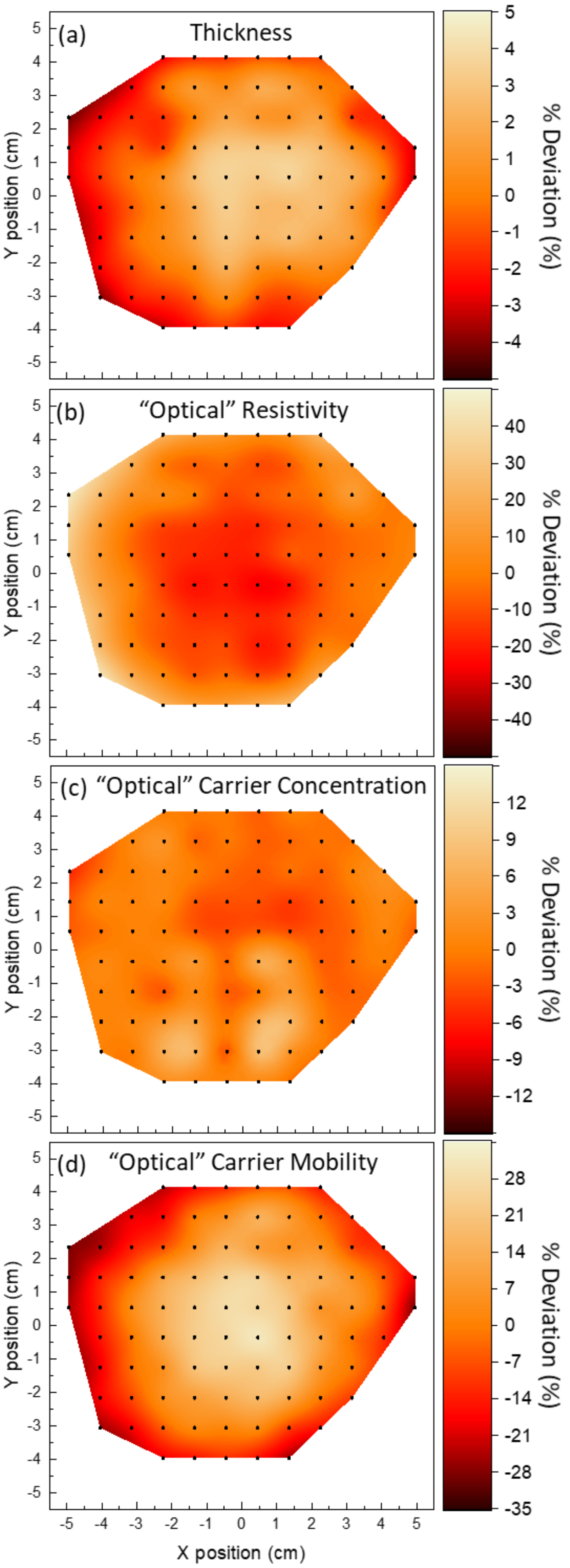

Figure S1. Colour map of the percentile deviation from the average centre value in (a) the film thickness, d_{film}, and the "optical" (b) resistivity, ρ_{opt}, (c) carrier concentration, N_{opt}, and (d) carrier mobility, μ_{opt}, of the ITO thin film coating across the 4″ Si wafer

Section B: Optical data

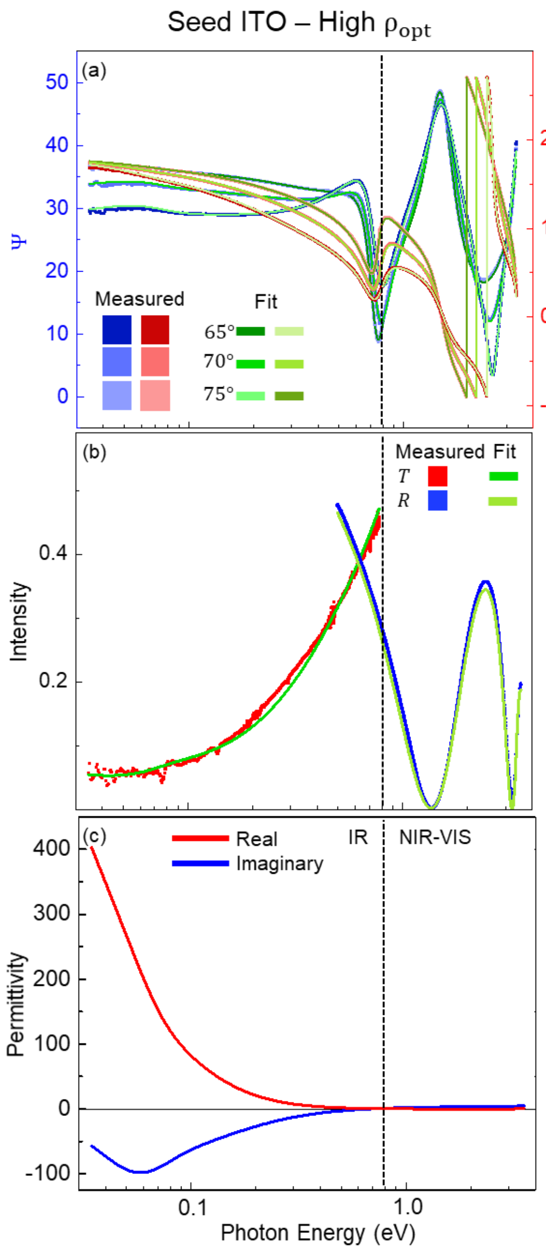

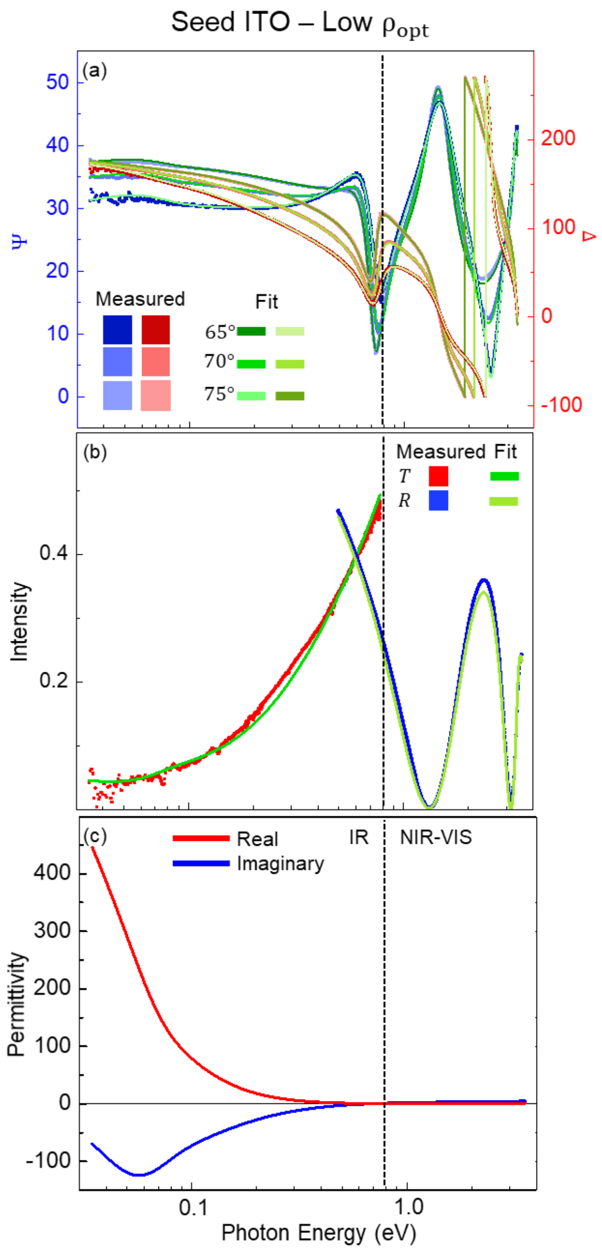

Figure S2. Fitting report for the **"1st" seed** ITO thin film with a resistivity at the **upper end** of the range exhibited by the seed films selected for further processing: (a) $\Psi(E)$ (blue squares) and $\Delta(E)$ (red squares) at incident angles of 55° (darker shade), 60° (normal shade) and 65° (lighter shade). (b) The corresponding IR transmission, $T_{IR}(E)$, (blue squares) and visible reflection, $R_{VIS}(E)$, (red squares). In (a) and (b), the solid green lines indicate the fit to each collected data set. (c) The corresponding, as-fit, real and imaginary parts of the complex permittivity, $\tilde{\varepsilon}(E) = \varepsilon_1(E) + i\varepsilon_2(E)$, (solid red and blue lines, respectively). Note the logarithmic scale in the x-axis of (a), (b) and (c). The vertical dashed black line indicates the boundary between the IR and NIR-VIS-UV ranges covered by the two ellipsometers.

Figure S3. Fitting report for the **"2nd" seed** ITO thin film with a resistivity at the **lower end** of the range exhibited by the seed films selected for further processing: (a) $\Psi(E)$ (blue squares) and $\Delta(E)$ (red squares) at incident angles of 55° (darker shade), 60° (normal shade) and 65° (lighter shade). (b) The corresponding IR transmission, $T_{IR}(E)$, (blue squares) and visible reflection, $R_{VIS}(E)$, (red squares). In (a) and (b), the solid green lines indicate the fit to each collected data set. (c) The corresponding, as-fit, real and imaginary parts of the complex permittivity, $\tilde{\varepsilon}(E) = \varepsilon_1(E) + i\varepsilon_2(E)$, (solid red and blue lines, respectively). Note the logarithmic scale in the x-axis of (a), (b) and (c). The vertical dashed black line indicates the boundary between the IR and NIR-VIS-UV ranges covered by the two ellipsometers.

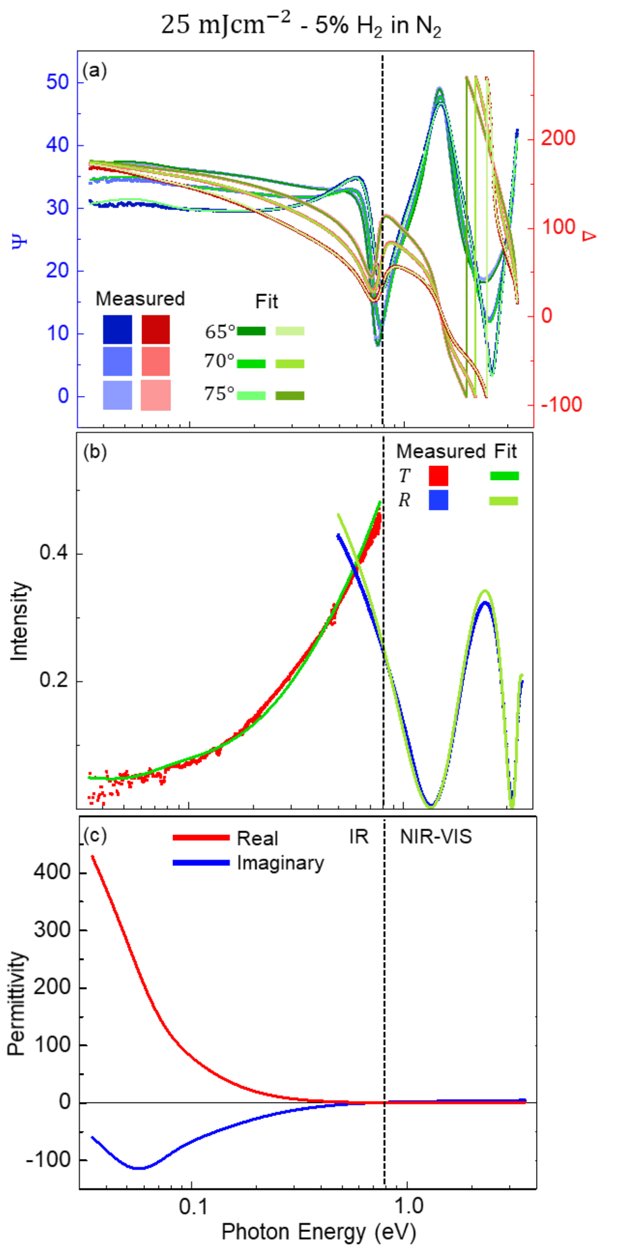

Figure S4. Fitting report for the ITO thin film: subject to single-pulse ReLA at **25 mJcm^{-2}** within a 100 psig pressured atmosphere of **5% H_2 in N_2**: (a) $\Psi(E)$ (blue squares) and $\Delta(E)$ (red squares) at incident angles of 55° (darker shade), 60° (normal shade) and 65° (lighter shade). (b) The corresponding IR transmission, $T_{IR}(E)$, (blue squares) and visible reflection, $R_{VIS}(E)$, (red squares). In (a) and (b), the solid green lines indicate the fit to each collected data set. (c) The corresponding, as-fit, real and imaginary parts of the complex permittivity, $\tilde{\varepsilon}(E) = \varepsilon_1(E) + i\varepsilon_2(E)$, (solid red and blue lines, respectively). Note the logarithmic scale in the x-axis of (a), (b) and (c). The vertical dashed black line indicates the boundary between the IR and NIR-VIS-UV ranges covered by the two ellipsometers.

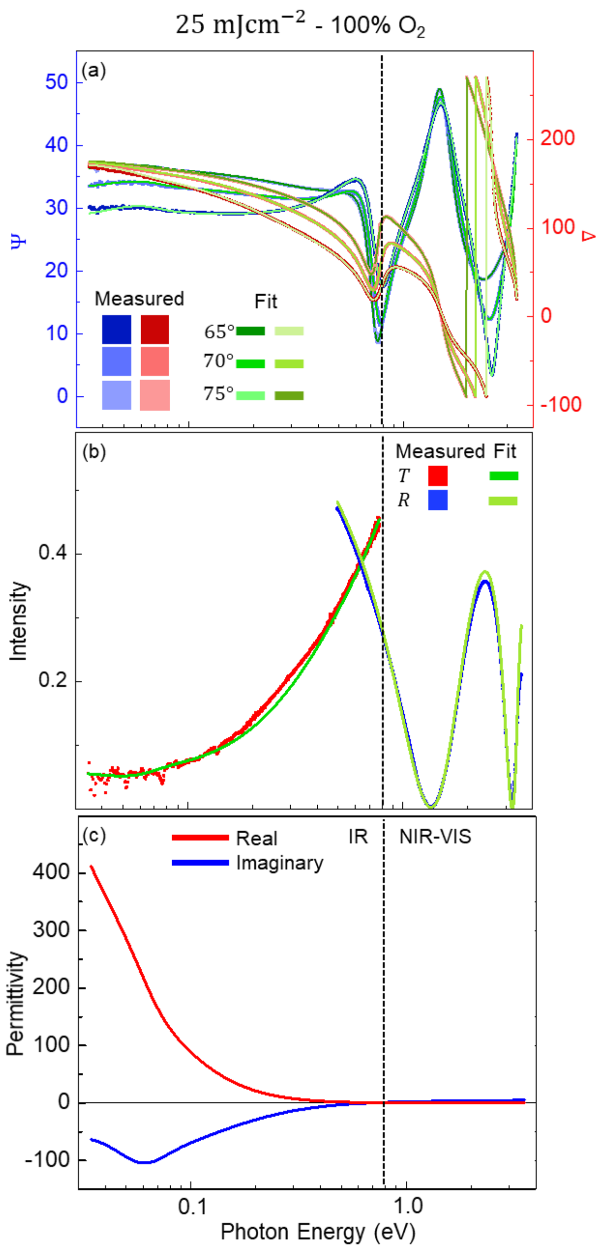

Figure S5. Fitting report for the ITO thin film: subject to single-pulse ReLA at **25 mJcm^{-2}** within a 100 psig pressured atmosphere of **100% O_2**: (a) $\Psi(E)$ (blue squares) and $\Delta(E)$ (red squares) at incident angles of 55° (darker shade), 60° (normal shade) and 65° (lighter shade). (b) The corresponding IR transmission, $T_{IR}(E)$, (blue squares) and visible reflection, $R_{VIS}(E)$, (red squares). In (a) and (b), the solid green lines indicate the fit to each collected data set. (c) The corresponding, as-fit, real and imaginary parts of the complex permittivity, $\tilde{\varepsilon}(E) = \varepsilon_1(E) + i\varepsilon_2(E)$, (solid red and blue lines, respectively). Note the logarithmic scale in the x-axis of (a), (b) and (c). The vertical dashed black line indicates the boundary between the IR and NIR-VIS-UV ranges covered by the two ellipsometers.

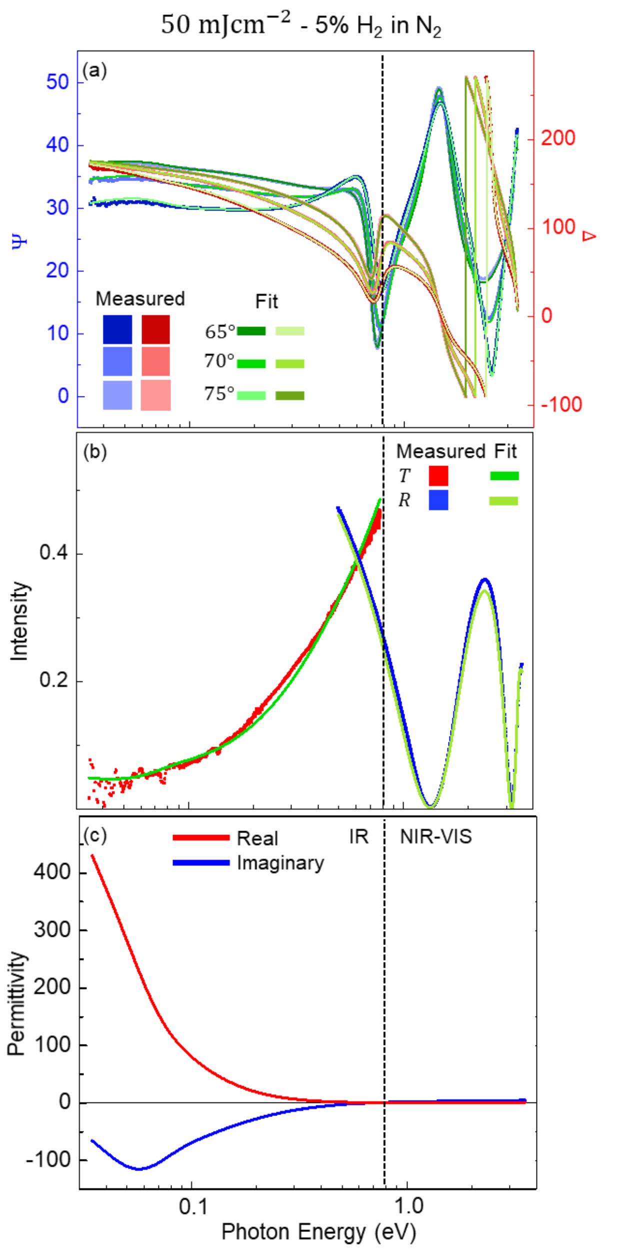

Figure S6. Fitting report for the ITO thin film: subject to single-pulse ReLA at **50 mJcm^{-2}** within a 100 psig pressured atmosphere of **5% H_2 in N_2**: (a) $\Psi(E)$ (blue squares) and $\Delta(E)$ (red squares) at incident angles of 55° (darker shade), 60° (normal shade) and 65° (lighter shade). (b) The corresponding IR transmission, $T_{IR}(E)$, (blue squares) and visible reflection, $R_{VIS}(E)$, (red squares). In (a) and (b), the solid green lines indicate the fit to each collected data set. (c) The corresponding, as-fit, real and imaginary parts of the complex permittivity, $\tilde{\varepsilon}(E) = \varepsilon_1(E) + i\varepsilon_2(E)$, (solid red and blue lines, respectively). Note the logarithmic scale in the x-axis of (a), (b) and (c). The vertical dashed black line indicates the boundary between the IR and NIR-VIS-UV ranges covered by the two ellipsometers.

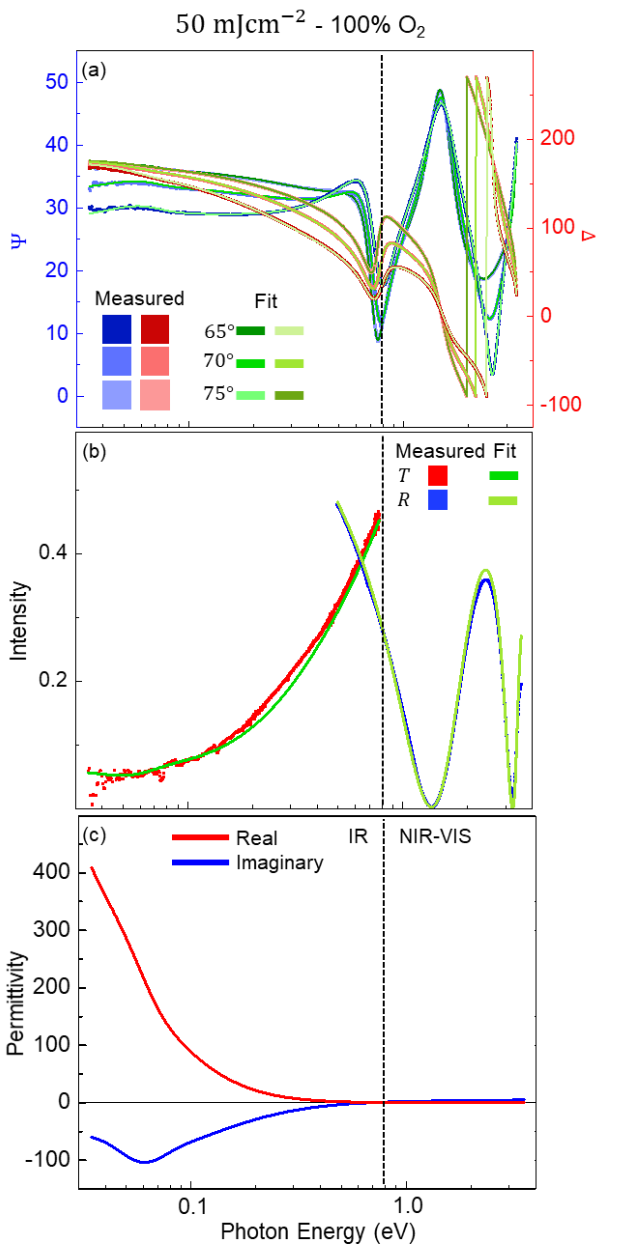

Figure S7. Fitting report for the ITO thin film: subject to single-pulse ReLA at **50mJcm^{-2}** within a 100 psig pressured atmosphere of **100% O_2**: (a) $\Psi(E)$ (blue squares) and $\Delta(E)$ (red squares) at incident angles of 55° (darker shade), 60° (normal shade) and 65° (lighter shade). (b) The corresponding IR transmission, $T_{IR}(E)$, (blue squares) and visible reflection, $R_{VIS}(E)$, (red squares). In (a) and (b), the solid green lines indicate the fit to each collected data set. (c) The corresponding, as-fit, real and imaginary parts of the complex permittivity, $\tilde{\varepsilon}(E) = \varepsilon_1(E) + i\varepsilon_2(E)$, (solid red and blue lines, respectively). Note the logarithmic scale in the x-axis of (a), (b) and (c). The vertical dashed black line indicates the boundary between the IR and NIR-VIS-UV ranges covered by the two ellipsometers.

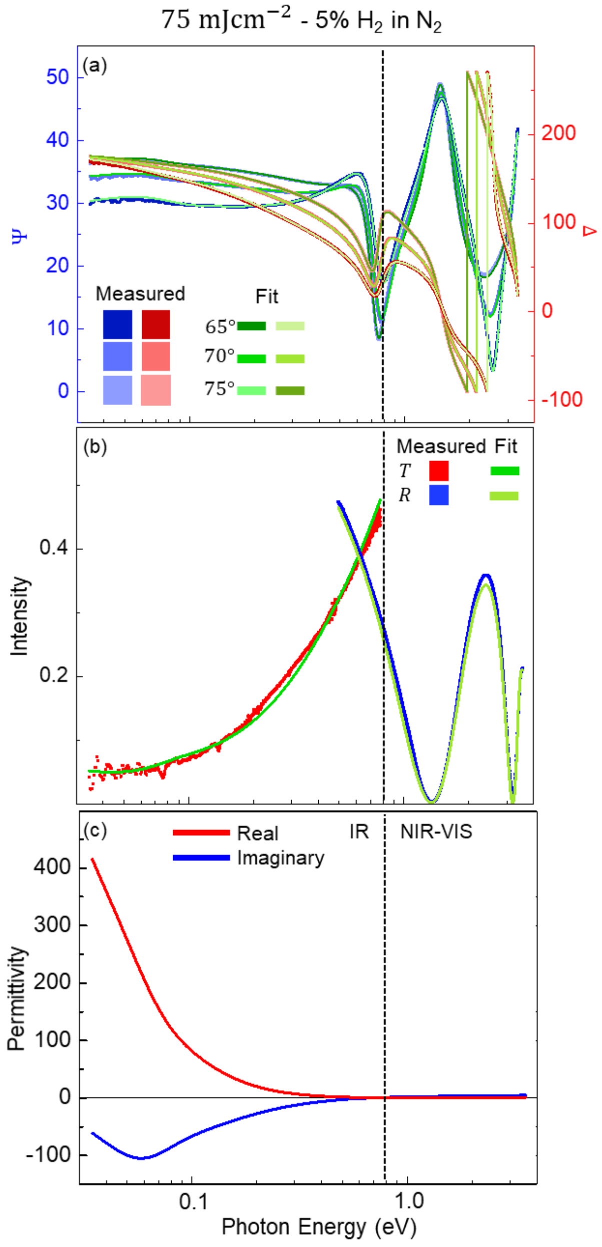

Figure S8. Fitting report for the ITO thin film: subject to single-pulse ReLA at **75 mJcm^{-2}** within a 100 psig pressured atmosphere of **5% H_2 in N_2**: (a) $\Psi(E)$ (blue squares) and $\Delta(E)$ (red squares) at incident angles of 55° (darker shade), 60° (normal shade) and 65° (lighter shade). (b) The corresponding IR transmission, $T_{IR}(E)$, (blue squares) and visible reflection, $R_{VIS}(E)$, (red squares). In (a) and (b), the solid green lines indicate the fit to each collected data set. (c) The corresponding, as-fit, real and imaginary parts of the complex permittivity, $\tilde{\varepsilon}(E) = \varepsilon_1(E) + i\varepsilon_2(E)$, (solid red and blue lines, respectively). Note the logarithmic scale in the x-axis of (a), (b) and (c). The vertical dashed black line indicates the boundary between the IR and NIR-VIS-UV ranges covered by the two ellipsometers.

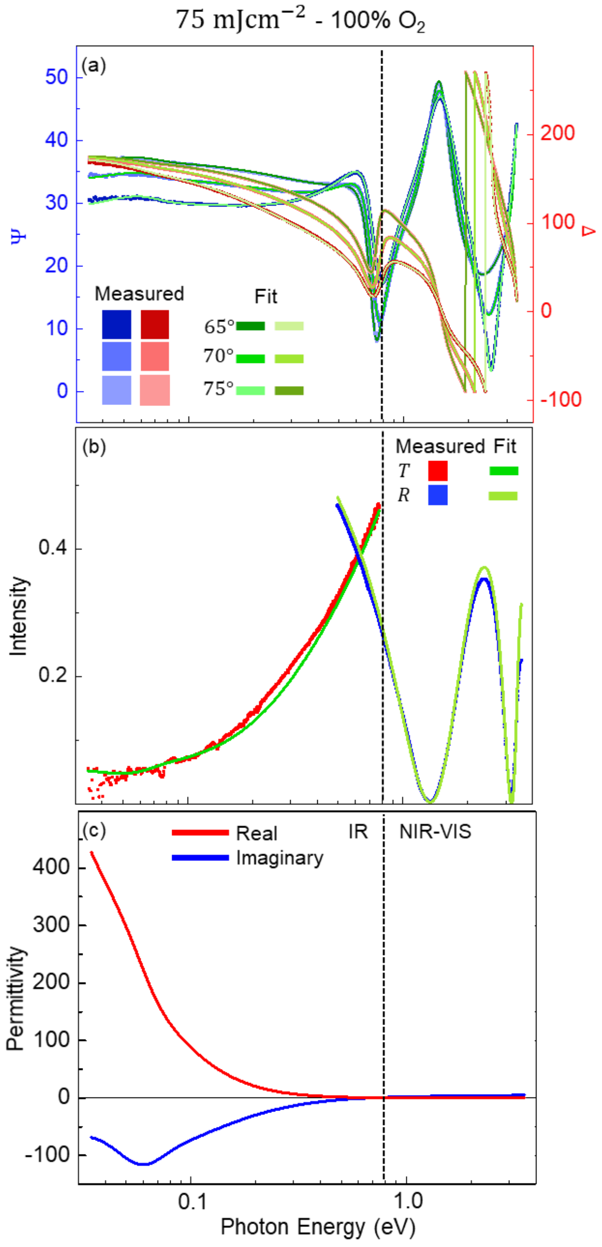

Figure S9. Fitting report for the ITO thin film: subject to single-pulse ReLA at **75 mJcm^{-2}** within a 100 psig pressured atmosphere of **100% O_2**: (a) $\Psi(E)$ (blue squares) and $\Delta(E)$ (red squares) at incident angles of 55° (darker shade), 60° (normal shade) and 65° (lighter shade). (b) The corresponding IR transmission, $T_{IR}(E)$, (blue squares) and visible reflection, $R_{VIS}(E)$, (red squares). In (a) and (b), the solid green lines indicate the fit to each collected data set. (c) The corresponding, as-fit, real and imaginary parts of the complex permittivity, $\tilde{\varepsilon}(E) = \varepsilon_1(E) + i\varepsilon_2(E)$, (solid red and blue lines, respectively). Note the logarithmic scale in the x-axis of (a), (b) and (c). The vertical dashed black line indicates the boundary between the IR and NIR-VIS-UV ranges covered by the two ellipsometers.

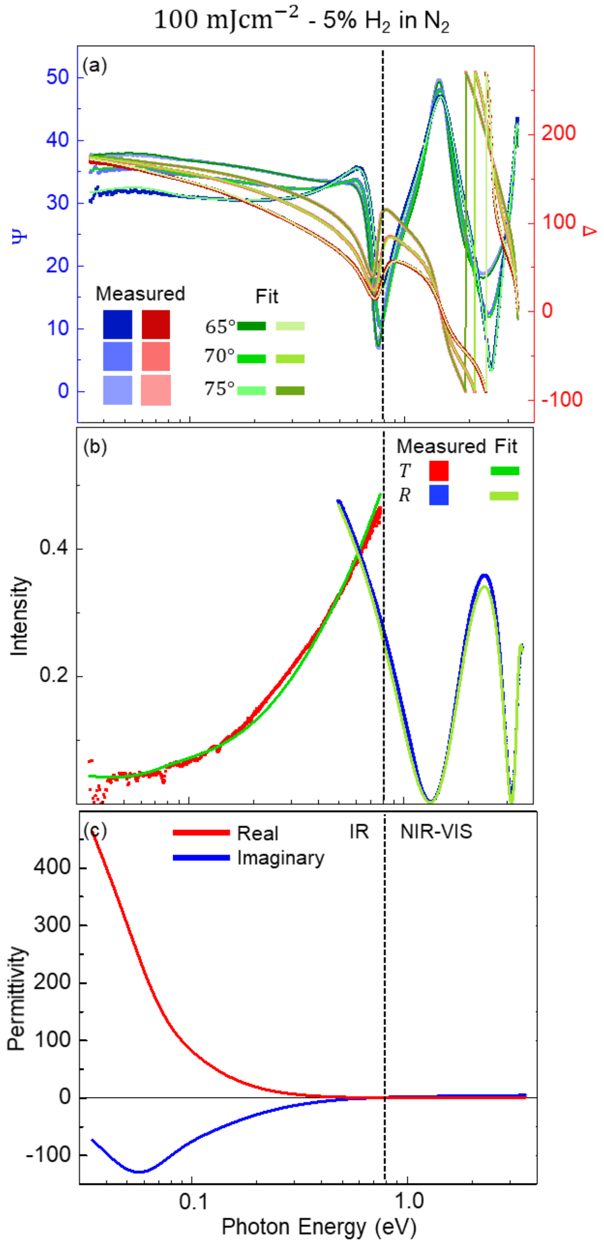

Figure S10. Fitting report for the ITO thin film: subject to single-pulse ReLA at **100 mJcm^{-2}** within a 100 psig pressured atmosphere of **5% H_2 in N_2**: (a) $\Psi(E)$ (blue squares) and $\Delta(E)$ (red squares) at incident angles of 55° (darker shade), 60° (normal shade) and 65° (lighter shade). (b) The corresponding IR transmission, $T_{IR}(E)$, (blue squares) and visible reflection, $R_{VIS}(E)$, (red squares). In (a) and (b), the solid green lines indicate the fit to each collected data set. (c) The corresponding, as-fit, real and imaginary parts of the complex permittivity, $\tilde{\varepsilon}(E) = \varepsilon_1(E) + i\varepsilon_2(E)$, (solid red and blue lines, respectively). Note the logarithmic scale in the x-axis of (a), (b) and (c). The vertical dashed black line indicates the boundary between the IR and NIR-VIS-UV ranges covered by the two ellipsometers.

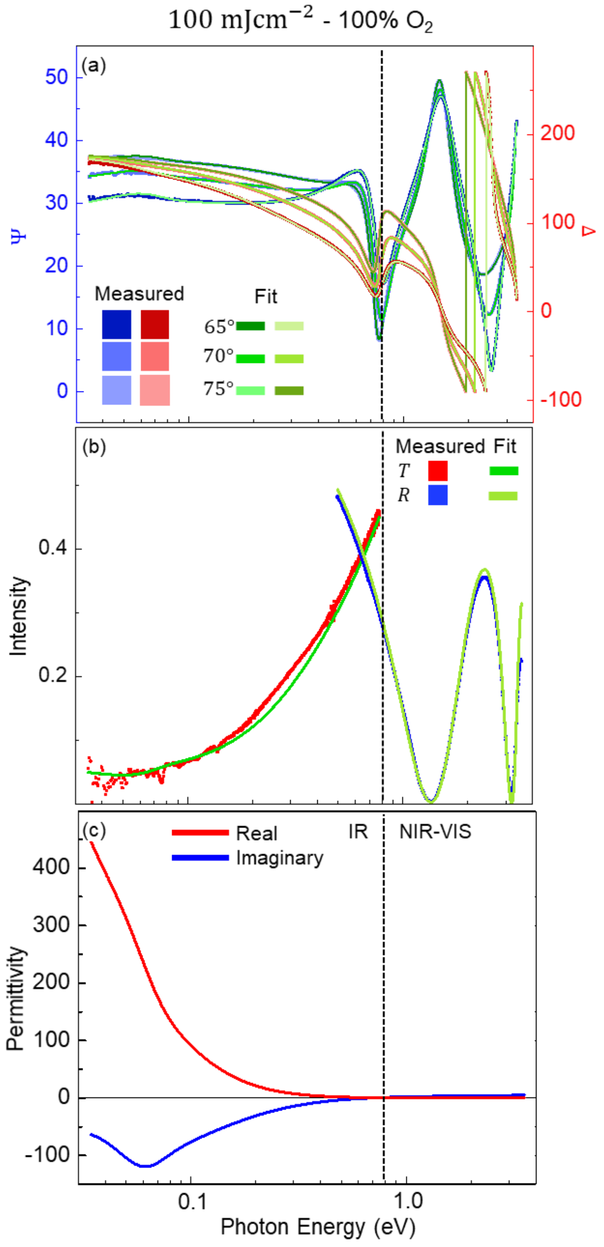

Figure S11. Fitting report for the ITO thin film: subject to single-pulse ReLA at **100 mJcm^{-2}** within a 100 psig pressured atmosphere of **100% O_2**: (a) $\Psi(E)$ (blue squares) and $\Delta(E)$ (red squares) at incident angles of 55° (darker shade), 60° (normal shade) and 65° (lighter shade). (b) The corresponding IR transmission, $T_{IR}(E)$, (blue squares) and visible reflection, $R_{VIS}(E)$, (red squares). In (a) and (b), the solid green lines indicate the fit to each collected data set. (c) The corresponding, as-fit, real and imaginary parts of the complex permittivity, $\tilde{\varepsilon}(E) = \varepsilon_1(E) + i\varepsilon_2(E)$, (solid red and blue lines, respectively). Note the logarithmic scale in the x-axis of (a), (b) and (c). The vertical dashed black line indicates the boundary between the IR and NIR-VIS-UV ranges covered by the two ellipsometers.

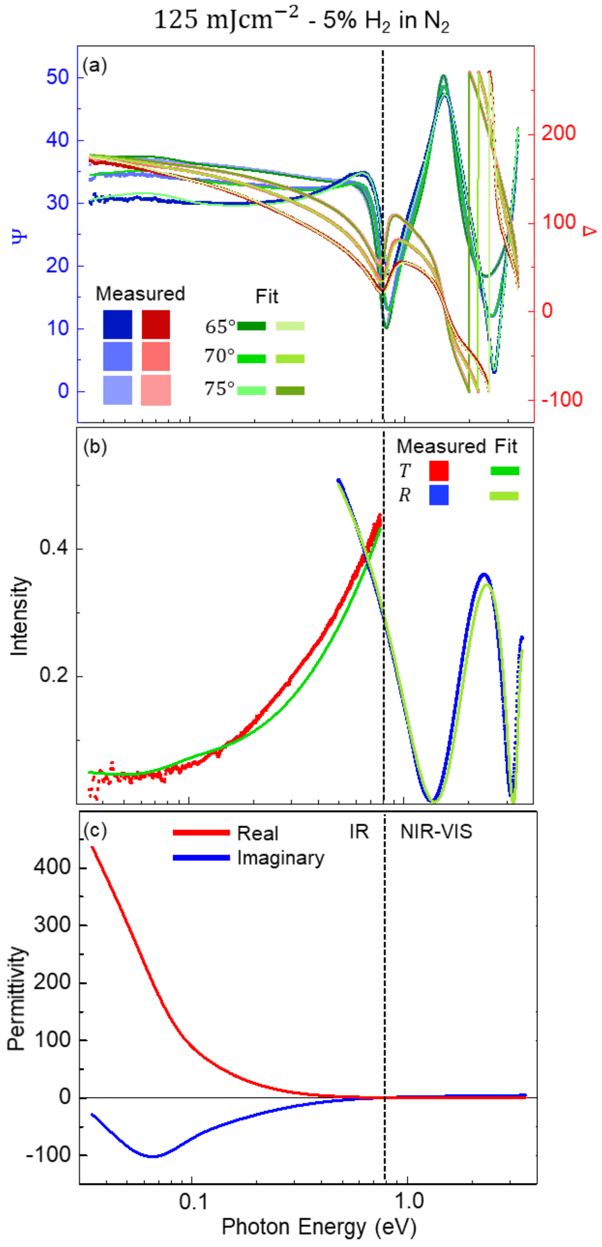

Figure S12. Fitting report for the ITO thin film: subject to single-pulse ReLA at **125 mJcm^{-2}** within a 100 psig pressured atmosphere of **5% H_2 in N_2**: (a) $\Psi(E)$ (blue squares) and $\Delta(E)$ (red squares) at incident angles of 55° (darker shade), 60° (normal shade) and 65° (lighter shade). (b) The corresponding IR transmission, $T_{IR}(E)$, (blue squares) and visible reflection, $R_{VIS}(E)$, (red squares). In (a) and (b), the solid green lines indicate the fit to each collected data set. (c) The corresponding, as-fit, real and imaginary parts of the complex permittivity, $\tilde{\varepsilon}(E) = \varepsilon_1(E) + i\varepsilon_2(E)$, (solid red and blue lines, respectively). Note the logarithmic scale in the x-axis of (a), (b) and (c). The vertical dashed black line indicates the boundary between the IR and NIR-VIS-UV ranges covered by the two ellipsometers.

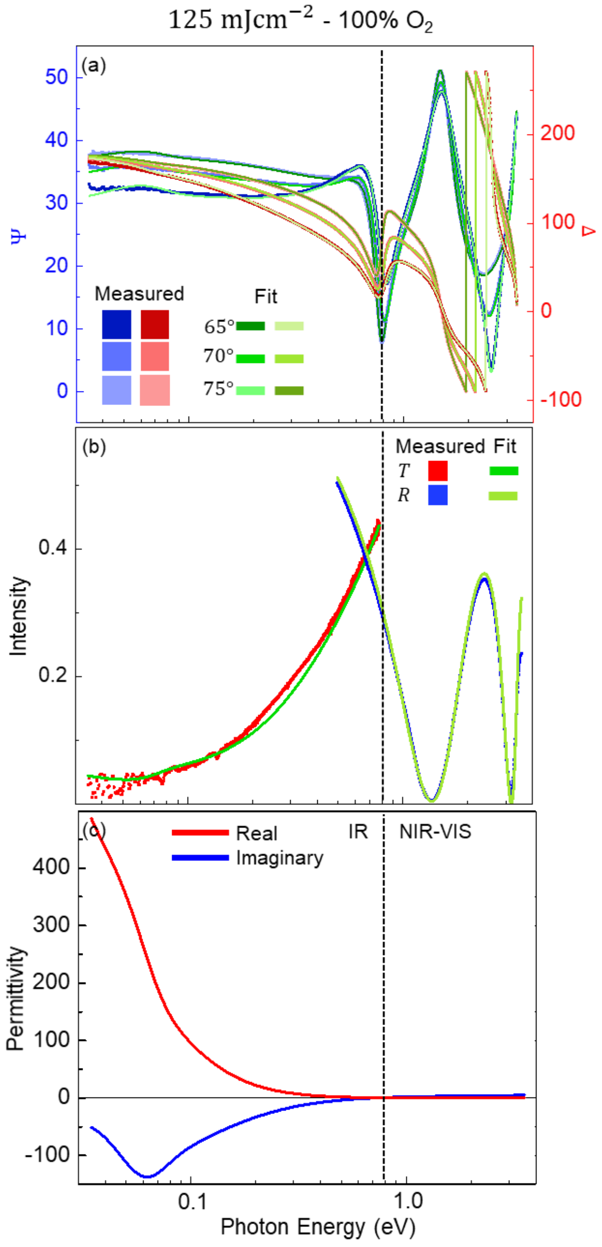

Figure S13. Fitting report for the ITO thin film: subject to single-pulse ReLA at **125 mJcm^{-2}** within a 100 psig pressured atmosphere of **100% O_2**: (a) $\Psi(E)$ (blue squares) and $\Delta(E)$ (red squares) at incident angles of 55° (darker shade), 60° (normal shade) and 65° (lighter shade). (b) The corresponding IR transmission, $T_{IR}(E)$, (blue squares) and visible reflection, $R_{VIS}(E)$, (red squares). In (a) and (b), the solid green lines indicate the fit to each collected data set. (c) The corresponding, as-fit, real and imaginary parts of the complex permittivity, $\tilde{\varepsilon}(E) = \varepsilon_1(E) + i\varepsilon_2(E)$, (solid red and blue lines, respectively). Note the logarithmic scale in the x-axis of (a), (b) and (c). The vertical dashed black line indicates the boundary between the IR and NIR-VIS-UV ranges covered by the two ellipsometers.

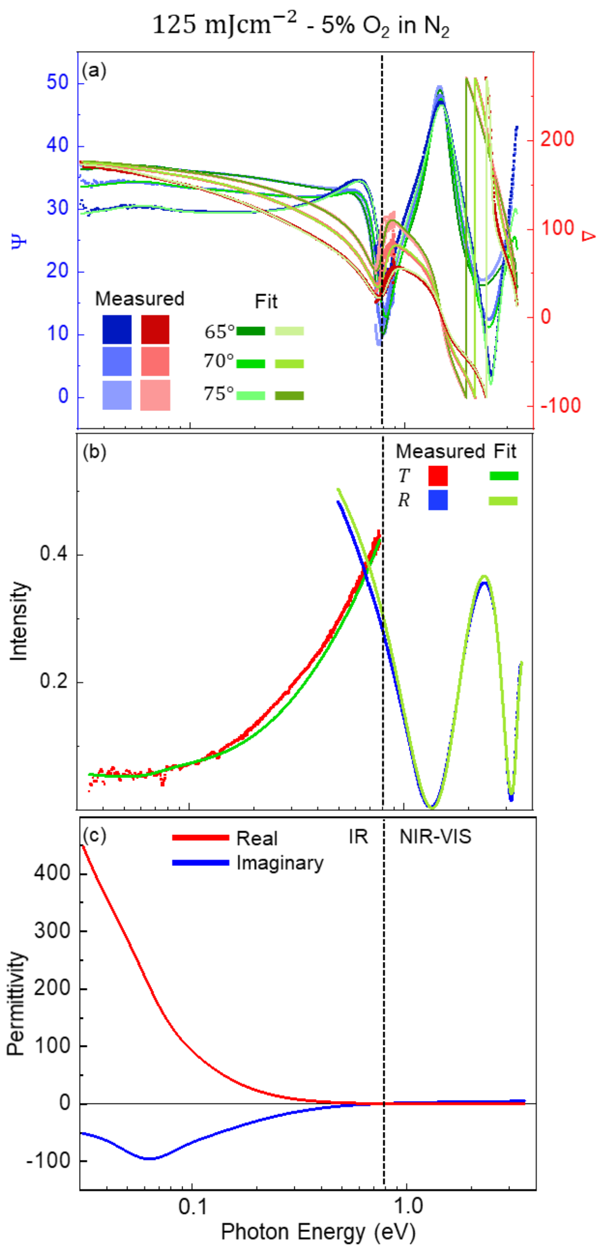

Figure S14. Fitting report for the ITO thin film: subject to single-pulse ReLA at **125 mJcm^{-2}** within a 100 psig pressured atmosphere of **5% O_2 in N_2**: (a) Ψ(E) (blue squares) and Δ(E) (red squares) at incident angles of 55° (darker shade), 60° (normal shade) and 65° (lighter shade). (b) The corresponding IR transmission, $T_{IR}(E)$, (blue squares) and visible reflection, $R_{VIS}(E)$, (red squares). In (a) and (b), the solid green lines indicate the fit to each collected data set. (c) The corresponding, as-fit, real and imaginary parts of the complex permittivity, $\tilde{\varepsilon}(E) = \varepsilon_1(E) + i\varepsilon_2(E)$, (solid red and blue lines, respectively). Note the logarithmic scale in the x-axis of (a), (b) and (c). The vertical dashed black line indicates the boundary between the IR and NIR-VIS-UV ranges covered by the two ellipsometers.

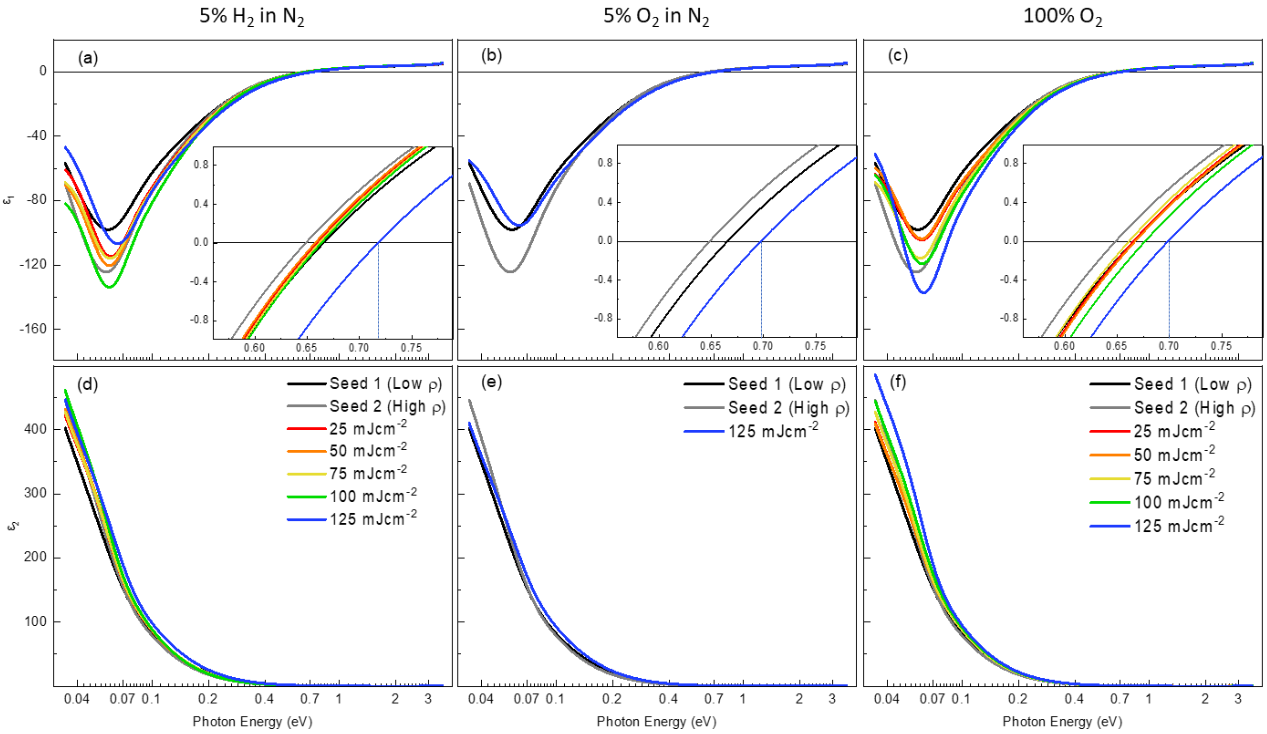

Figure S15. (a-c) Real and (d-f) imaginary parts of the complex permittivity, $\tilde{\varepsilon}(E) = \varepsilon_1(E) + i\varepsilon_2(E)$, reported for the seed ITO thin films (black and grey lines) and those: subject to single-pulse ReLA at $25 - 125$ mJcm^{-2} (red to blue lines in rainbow order) within a 100 psig pressured atmosphere of (a, d) 5% H_2 in N_2, (b, e) 5% O_2 in N_2, and (c, f) 100% O_2, respectively. The inset in (a) and (b) present a close-up of the point where $\varepsilon_1(E)$ crosses the x-axis. The blue vertical lines indicate the plasma energy of the films annealed at 125 mJcm^{-2}. Note the logarithmic scale in the x-axis of (a), (b) and (c).

Parameterised Results

Table S1. Numerical values for each parameter used to calculate the optical constants of the seed ITO films and the ITO film subject to LA at 125 $mJcm^{-2}$ into an ambient environment of 5% O_2 in N_2.

**Environment**		**n/a – Seed ITO (Low ρ_{opt})**		**n/a – Seed ITO (High ρ_{opt})**		**5% O_2 in N_2**	
**Fluence**	**$mJcm^{-2}$**					**124**	**2**
**Ellipsometrically Determined Parameters**							
MSE		8.139		8.303		9.959	
**Parameter**	**Unit**	**Value**	**Error**	**Value**	**Error**	**Value**	**Error**
Thickness	nm	127.58	0.04	131.36	0.05	129.95	0.06
Surface Roughness	nm	5.09	0.07	5.32	0.08	5.35	0.07
High-Frequency Permittivity		3.365	0.003	3.287	0.003	3.293	0.004
"Optical" Resistivity	$\times 10^{-4}$ Ωcm	5.40	0.01	4.89	0.01	4.94	0.01
"Optical" Mean Free Time	fs	4.863	0.006	5.771	0.009	4.945	0.008
Inhomogeneity	%	10.9	0.2	12.5	0.2	11.2	0.3
**1st Oscillator (Gaussian)**							
**Parameter**	**Unit**	**Value**	**Error**	**Value**	**Error**	**Value**	**Error**
Amplitude		42	1	42	1		
Broadening	eV	0.0352	0.0008	0.0294	0.0007		
Energy	eV	0.045	– –	0.045	– –	0.045	– –
**2nd Oscillator (Lorentz)**							
**Parameter**	**Unit**	**Value**	**Error**	**Value**	**Error**	**Value**	**Error**
Amplitude		7.46	0.05	9.51	0.07		
Broadening	eV	0.410	0.002	0.346	0.002		
Energy	eV	4.229	– –	4.229	– –	4.229	– –
**Non-Optically Measured Parameters**							
**Parameter**	**Unit**	**Value**	**Error**	**Value**	**Error**	**Value**	**Error**
DC Resistivity (Van der Pauw)	$\times 10^{-4}$ Ωcm	4.27	0.04	4.87	0.05	4.40	0.02
DC Resistivity (4pp)	$\times 10^{-4}$ Ωcm	4.2	0.1	5.0	0.3	4.2	0.3
"Hall" Carrier Concentration	$\times 10^{20}$ cm^{-3}	4.3	0.3	4.8	0.3	5.5	0.9
**Calculated Parameters**							
**Parameter**	**Unit**	**Value**	**Error**	**Value**	**Error**	**Value**	**Error**
Effective carrier mass ratio*		0.355	0.007	0.348	0.007	0.37	0.02
"Optical" Carrier Concentration	$\times 10^{20}$ cm^{-3}	4.8	0.1	4.38	0.09	5.3	0.3
"Optical" Carrier Mobility	cm^2/Vs	24.1	0.5	29.1	0.6	24	1
"Hall" Carrier Mobility	cm^2/Vs	26.99	0.02	33.71	0.02	25.8	0.2
Screened plasma energy	eV	0.6655	0.0003	0.6484	0.0003	0.6972	0.0009

* The effective carrier mass ratio is calculated from the Hall carrier concentration by accounting for non-parabolicity in the conduction band.[6]

Table S2. Numerical values for each parameter used to calculate the optical constants of the ITO films subject to LA in 5% H_2 in N_2.

**Environment**		**5% H_2 in N_2**		**5% H_2 in N_2**		**5% H_2 in N_2**		**5% H_2 in N_2**		**5% H_2 in N_2**	
**Fluence**	**$mJcm^{-2}$**	**25.1**	**0.3**	**49.7**	**0.8**	**75**	**1**	**100**	**2**	**124**	**2**
**Ellipsometrically Determined Parameters**											
MSE		14.91		9.09		9.35		9.30		14.66	
**Parameter**	**Unit**	**Value**	**Error**	**Value**	**Error**	**Value**	**Error**	**Value**	**Error**	**Value**	**Error**
Thickness	nm	130.76	0.07	130.26	0.04	130.27	0.04	131.33	0.04	126.49	0.09
Surface Roughness	nm	4.1	0.1	5.19	0.07	5.00	0.07	5.16	0.07	6.0	0.1
High-Frequency Permittivity		3.404	0.005	3.319	0.003	3.336	0.003	3.311	0.003	3.368	0.007
"Optical" Resistivity	$\times 10^{-4}$ Ωcm	5.29	0.02	5.09	0.01	5.16	0.01	4.74	0.01	5.22	0.02
"Optical" Mean Free Time	fs	5.17	0.01	5.420	0.008	5.274	0.008	5.748	0.009	4.41	0.01
Inhomogeneity	%	2.9	0.3	11.0	0.2	9.9	0.2	8.7	0.2	9.5	0.5
**1st Oscillator (Gaussian)**											
**Parameter**	**Unit**	**Value**	**Error**	**Value**	**Error**	**Value**	**Error**	**Value**	**Error**	**Value**	**Error**
Amplitude		42	1	42	1	42	1	42	1	42	1
Broadening	eV	0.0294	0.0007	0.032	0.001	0.0303	0.0007	0.0314	0.0007	0.0307	0.0007
Energy	eV	0.045	--	0.045	--	0.045	--	0.045	--	0.045	--
**2nd Oscillator (Lorentz)**											
**Parameter**	**Unit**	**Value**	**Error**	**Value**	**Error**	**Value**	**Error**	**Value**	**Error**	**Value**	**Error**
Amplitude		7.55	0.05	7.46	0.05	9.51	0.07	6.84	0.08	8.60	0.06
Broadening	eV	0.407	0.002	0.410	0.002	0.346	0.002	0.417	0.004	0.368	0.002
Energy	eV	4.229	--	4.229	--	4.229	--	4.229	--	4.229	--
**Non-Optically Measured Parameters**											
**Parameter**	**Unit**	**Value**	**Error**	**Value**	**Error**	**Value**	**Error**	**Value**	**Error**	**Value**	**Error**
DC Resistivity (Van der Pauw)	$\times 10^{-4}$ Ωcm	4.69	0.02	4.42	0.07	4.59	0.07	4.15	0.07	4.4	0.2
DC Resistivity (4pp)	$\times 10^{-4}$ Ωcm	4.9	0.3	4.7	0.3	4.7	0.3	4.1	0.5	3.8	0.3
"Hall" Carrier Concentration	$\times 10^{20}$ cm^{-3}	4.8	0.3	4.4	0.2	4.6	0.2	4.4	0.3	5.7	0.7
**Calculated Parameters**											
**Parameter**	**Unit**	**Value**	**Error**	**Value**	**Error**	**Value**	**Error**	**Value**	**Error**	**Value**	**Error**
Effective carrier mass ratio*		0.356	0.009	0.349	0.005	0.353	0.005	0.349	0.007	0.37	0.02
"Optical" Carrier Concentration	$\times 10^{20}$ cm^{-3}	4.6	0.1	4.43	0.07	4.56	0.07	4.6	0.1	5.7	0.2
"Optical" Carrier Mobility	cm^2/Vs	25.8	0.6	27.6	0.4	26.6	0.4	29.3	0.6	21.2	0.9
"Hall" Carrier Mobility	cm^2/Vs	27.759	0.007	32.41	0.02	29.46	0.02	34.21	0.04	24.6	0.1
Screened plasma energy	eV	0.6592	0.0001	0.6573	0.0003	0.6614	0.0002	0.6636	0.0004	0.7178	0.0004

* The effective carrier mass ratio is calculated from the Hall carrier concentration by accounting for non-parabolicity in the conduction band.[6]

Table S3. Numerical values for each parameter used to calculate the optical constants of the ITO films subject to LA in 100% O_2.

**Environment**		**100% O_2**		**100% O_2**		**100% O_2**		**100% O_2**		**100% O_2**	
**Fluence**	**$mJcm^{-2}$**	**25.1**	**0.3**	**49.7**	**0.8**	**75**	**1**	**100**	**2**	**124**	**2**
**Ellipsometrically Determined Parameters**											
MSE		14.91		9.09		9.35		9.30		14.66	
**Parameter**	**Unit**	**Value**	**Error**	**Value**	**Error**	**Value**	**Error**	**Value**	**Error**	**Value**	**Error**
Thickness	nm	128.88	0.04	127.64	0.04	130.27	0.04	129.95	0.05	131.14	0.05
Surface Roughness	nm	5.05	0.07	5.15	0.07	5.00	0.07	4.33	0.08	4.21	0.09
High-Frequency Permittivity		3.358	0.003	3.357	0.003	3.336	0.003	3.372	0.003	3.362	0.003
"Optical" Resistivity	$\times 10^{-4}$ Ωcm	5.38	0.01	5.43	0.01	5.16	0.01	5.05	0.01	4.83	0.01
"Optical" Mean Free Time	fs	4.955	0.006	4.865	0.007	5.274	0.008	5.138	0.008	5.156	0.009
Inhomogeneity	%	10.1	0.2	11.1	0.2	9.9	0.2	7.0	0.2	3.4	0.2
**1st Oscillator (Gaussian)**											
**Parameter**	**Unit**	**Value**	**Error**	**Value**	**Error**	**Value**	**Error**	**Value**	**Error**	**Value**	**Error**
Amplitude		42	1	42	1	42	1	42	1	42	1
Broadening	eV	0.0339	0.0007	0.0331	0.0007	0.0314	0.0007	0.0339	0.0007	0.0351	0.0006
Energy	eV	0.045	– –	0.045	– –	0.045	– –	0.045	– –	0.045	– –
**2nd Oscillator (Lorentz)**											
**Parameter**	**Unit**	**Value**	**Error**	**Value**	**Error**	**Value**	**Error**	**Value**	**Error**	**Value**	**Error**
Amplitude		7.68	0.05	7.55	0.05	8.30	0.06	8.44	0.06	8.75	0.07
Broadening	eV	0.398	0.002	0.407	0.002	0.375	0.002	0.362	0.002	0.344	0.002
Energy	eV	4.229	– –	4.229	– –	4.229	– –	4.229	– –	4.229	– –
**Non-Optically Measured Parameters**											
**Parameter**	**Unit**	**Value**	**Error**	**Value**	**Error**	**Value**	**Error**	**Value**	**Error**	**Value**	**Error**
DC Resistivity (Van der Pauw)	$\times 10^{-4}$ Ωcm	4.65	0.08	4.66	0.09	4.48	0.07	4.23	0.07	3.82	0.07
DC Resistivity (4pp)	$\times 10^{-4}$ Ωcm	5.2	0.3	5.3	0.3	4.9	0.3	4.7	0.2	4.0	0.2
"Hall" Carrier Concentration	$\times 10^{20}$ cm^{-3}	4.6	0.4	4.7	0.2	4.2	0.1	4.9	0.2	4.9	0.3
**Calculated Parameters**											
**Parameter**	**Unit**	**Value**	**Error**	**Value**	**Error**	**Value**	**Error**	**Value**	**Error**	**Value**	**Error**
Effective carrier mass ratio*		0.353	0.009	0.354	0.005	0.346	0.004	0.358	0.005	0.357	0.007
"Optical" Carrier Concentration	$\times 10^{20}$ cm^{-3}	4.7	0.1	4.75	0.07	4.52	0.05	4.89	0.07	5.1	0.1
"Optical" Carrier Mobility	cm^2/Vs	24.7	0.7	24.2	0.4	26.8	0.3	25.2	0.4	25.4	0.5
"Hall" Carrier Mobility	cm^2/Vs	29.21	0.04	28.75	0.02	33.11	0.02	29.97	0.02	33.47	0.04
Screened plasma energy	eV	0.6655	0.0003	0.6673	0.0001	0.6614	0.0002	0.6766	0.0003	0.6987	0.0002

* The effective carrier mass ratio is calculated from the Hall carrier concentration by accounting for non-parabolicity in the conduction band.[6]

Section C: Ellipsometric modelling

Due to the nature of the "inverse problem" of SE, where the sample properties cannot be directly calculated from the measured ellipsometric quantities but the ellipsometry quantities can be calculated from any given sample properties, the precise nature of the fitting is highly dependent on the sample of interest.[7]

Oscillator Functions

Lorentz functions produced the best fit when utilised for UV absorption tail.[8] They are described by Eq. (S1):

$$\tilde{\varepsilon}_{\mathrm{Lorentz}}(\mathrm{E}) = \frac{\mathrm{A_L}\gamma\mathrm{E_0}}{\mathrm{E_0^2} - \mathrm{E^2} - \mathrm{iE}\gamma} \quad \text{(S1)}$$

where A_L is the oscillator amplitude, E_0 is the centre energy and γ is the broadening of the oscillator. Gaussian functions produced the best fit when used for IR absorption centres.[9] They are described by Eqs. (S2) and (S3).

$$\tilde{\varepsilon}_{\mathrm{Guassian}}(\mathrm{E}) = \mathrm{A_L}\left\{\begin{array}{c}\left[\Gamma\left(\frac{\mathrm{E}-\mathrm{E_0}}{\sigma}\right)+\Gamma\left(\frac{\mathrm{E}+\mathrm{E_0}}{\sigma}\right)\right] \\ +\mathrm{i}\cdot\left(\exp\left[-\left(\frac{\mathrm{E}-\mathrm{E_0}}{\sigma}\right)^2\right]-\exp\left[-\left(\frac{\mathrm{E}+\mathrm{E_0}}{\sigma}\right)^2\right]\right)\end{array}\right\} \quad \text{(S2)}$$

where

$$\sigma = \frac{\gamma}{2\sqrt{\ln(2)}} \quad \text{(S3)}$$

and the function Γ is a convergence series used to ensure Kramers-Kronig consistency for the real permittivity.[9] The other parameters are defined above.

Justification of SE model

This section outlines the process used to fit SE measurements across the entire IR and NIR-VIS spectral range ($0.034 - 3.4$ eV) to extract the complex permittivity, $\tilde{\varepsilon}(E) = \varepsilon_1(E) + i\varepsilon_2(E)$, and optoelectronic properties of the ITO films. A model that considers both the intra- and inter-band absorption is required to fit the optical properties of ITO. To achieve this, we require a "generalised oscillator" model that describes the permittivity of a material as a summation of various individual oscillators.[7]

To fit the measurement with a generalised oscillator model, a summation is made of one Drude and one Lorentz oscillator in order to describe both the absorption due to free carriers (intra-band absorption) and inter-band transitions, respectively, as has been suggested by Uprety *et al.* for ITO films.[10] To improve the fit further, it was necessary to include an additional IR phonon absorption (described by a Gaussian oscillator) at ~0.045 eV. Due to amorphous structure of the ITO film and the screening of the peak by the free carrier absorption, the Sn-O, Sn-O-Sn, and In-O optical phonon modes cannot be individually resolved.[11] The Gaussian oscillator at ~0.045 eV can be said to represent a convolution of the peaks. The final step to accurately fit the NIR-VIS SE data was to include a layer of "optical" surface roughness, described by an effective medium approximation consisting of 50% of the ITO layer below and 50% air.

It is important to note that any fit can be achieved, given enough oscillators. "Over-fitting" of the data in this way can complicate the ability to extract useful information from the generalised oscillator model. Potential over-fitting of the data can be investigated *via* the parameter correlation,[7] a number between negative and positive unity assigned to each parameter pair where absolute values above 0.90 indicate that the variations in one parameter can be completely accounted for by variations in its correlated counterpart. The parameter correlation can be reduced, increasing the validity of the fit, by reducing the number of free parameters.[1] A general rule is any added complexity in the model (*i.e.,* introducing surface roughness or additional oscillators etc.) is valid if it produces a decrease in the mean-squared error (MSE), which defines how well the data is fit, by at least ~25%.[7] Exceptions to this rule can be made if the parameter is thought to improve the "realness" of the model.

To guarantee confidence in the physical reality of the extracted optical constants and optoelectronic parameters, these principles were followed during the optical modelling: ensuring that the utilised model was as simple as possible while producing a good fit to data, with only few and largely uncorrelated fitting parameters, and had been constructed following the guidance of literature. Where possible, the parameters have been verified with independent techniques. For example, the carrier transport properties have been independently measured via Hall Effect. Figure S16 compares the "Hall" (filled symbols) and "optical" (open symbols) (a) carrier concentration, N_{Hall} and N_{opt} respectively, and (b) mobility, μ_{Hall} and μ_{opt} respectively, for the seed ITO films (grey stars) and those subject to reactive laser annealing in 5% H_2 in N_2 (red circles), 5% O_2 in N_2 (blue squares), and 100% O_2 (green triangles) and between $25 - 125$ mJcm^{-2} in steps of 25 mJcm^{-2}. Further validation of the resistivity was achieved *via* four-point probe (Table S1-Table **S3**) and the gradient in the carrier concentration was independently measured with depth-profile energy-dispersive X-ray spectroscopy (Figure S20-Figure S22).

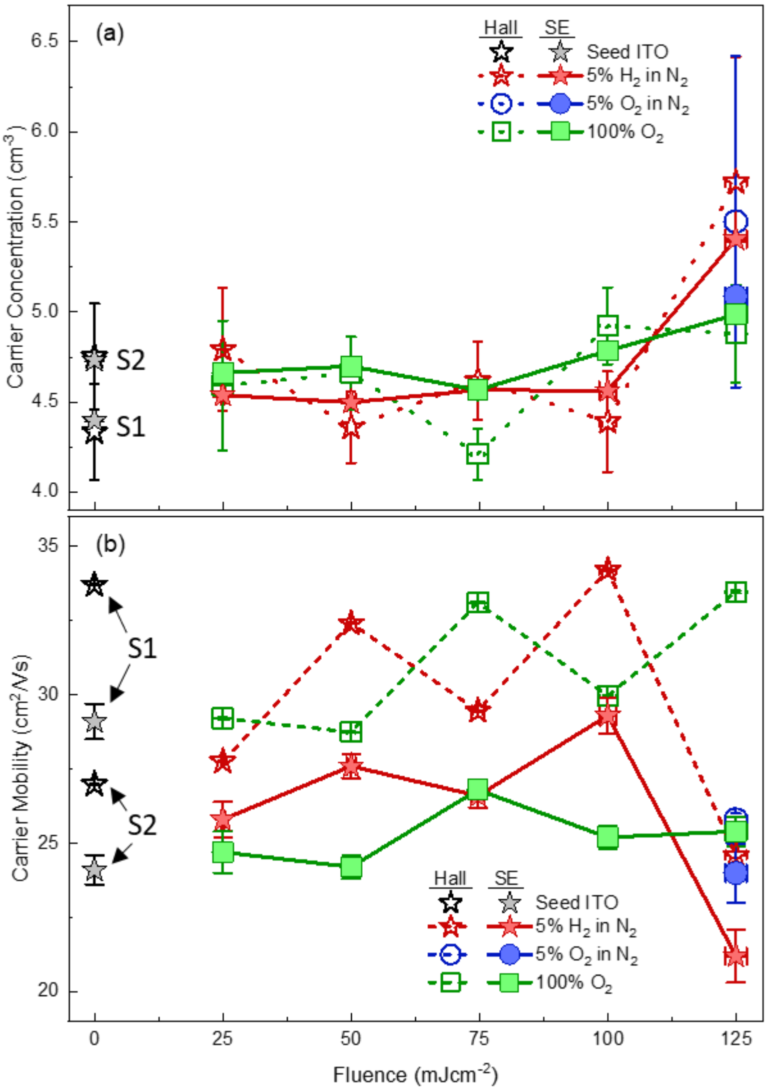

Figure S16. (a) "Hall" (open symbols, dashed lines) and "optical" (filled symbols, solid lines) carrier concentration, N_{Hall} and N_{opt} respectively, for the two seed ITO films (grey stars; S1 with higher resistivity and S2 with lower resistivity) and those subject to reactive laser annealing in 5% H_2 in N_2 (red circles), 5% O_2 in N_2 (blue squares), and 100% O_2 (green triangles) and between $25 - 125$ $mJcm^{-2}$ in steps of 25 $mJcm^{-2}$. (b) "Hall" (open symbols, dashed lines) and "optical" (filled symbols, solid lines) carrier mobility, μ_{Hall} and μ_{opt} respectively, seed ITO films (grey stars) and those subject to reactive laser annealing in 5% H_2 in N_2 (red circles), 5% O_2 in N_2 (blue squares), and 100% O_2 (green triangles) and between $25 - 125$ $mJcm^{-2}$ in steps of 25 $mJcm^{-2}$.

Section D: XRD data and analysis

XRD spectra for all ITO films

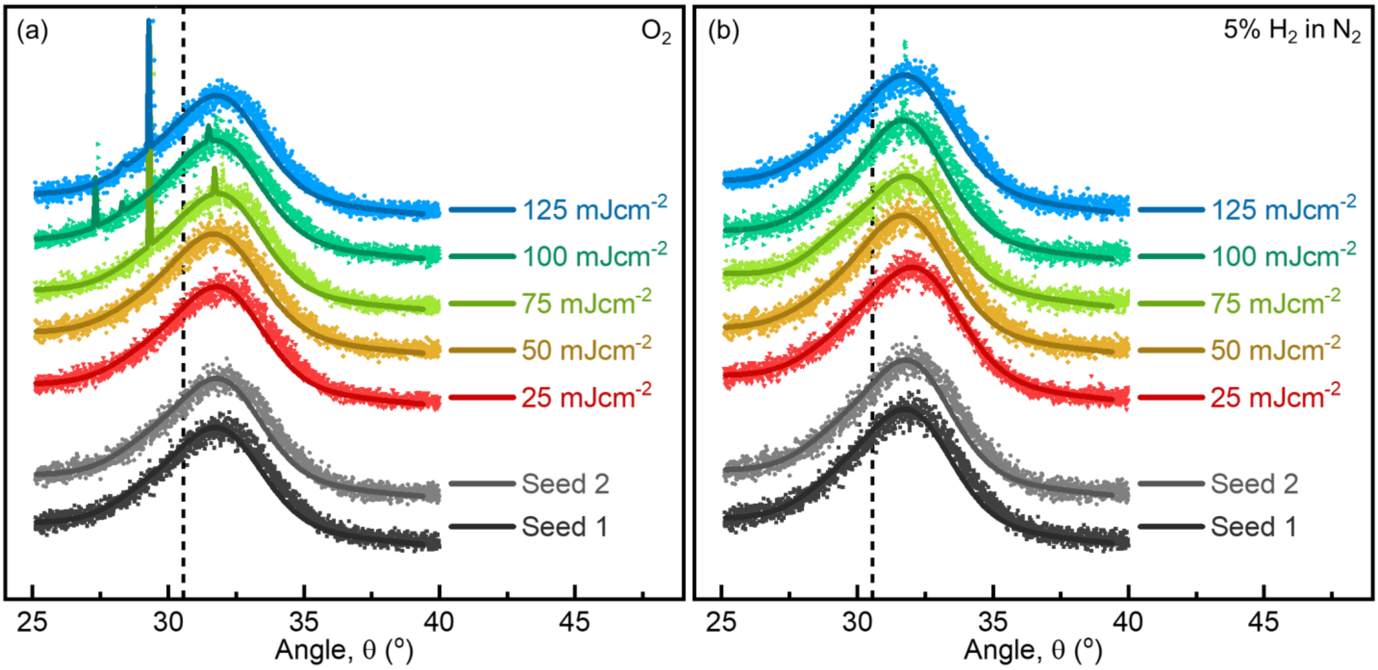

Figure S17. X-ray diffractograms for the two seed ITO films (dark-light grey squares; Seed 1 with higher resistivity and Seed 2 with lower resistivity) and those subject to reactive laser annealing in (a) 100% O_2 and (b) 5% H_2 in N_2 between $25 - 125$ mJcm^{-2} in steps of 25 mJcm^{-2} (red-blue squares). The fitting of two *pseudo*-Voigt functions to the asymmetric peak of each film is shown with the solid lines. To facilitate the peak fitting, the (100) Si peak at $32.9^0 - 33.1^0$ has been manually removed from all diffractograms.

XRD analysis

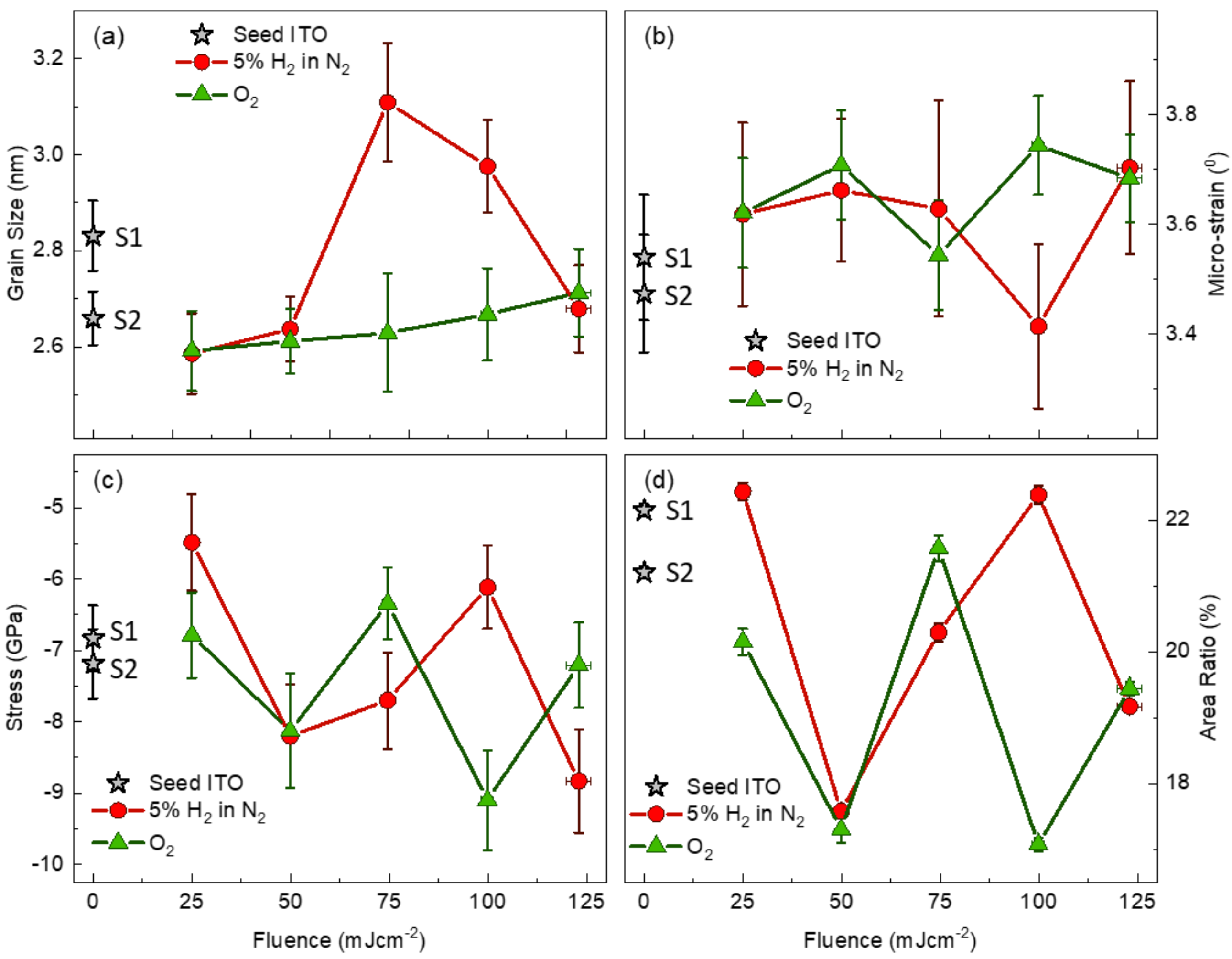

Figure S18. X-ray analysis results: (a) Grain size, (b) micro-strain, (c) stress, and (d) area ratio between the two *pseudo*-Voigt peaks for the two seed ITO films (grey stars; S1 with higher resistivity and S2 with lower resistivity) and those subject to single-pulse reactive laser annealing in O_2 (green triangles; dark green line) and 5% H_2 in N_2 (red circles; dark red line) at $25 - 125$ $mJcm^{-2}$ in steps of 25 $mJcm^{-2}$.

Section E: XPS and EDX data and Analysis

XPS Data and fits

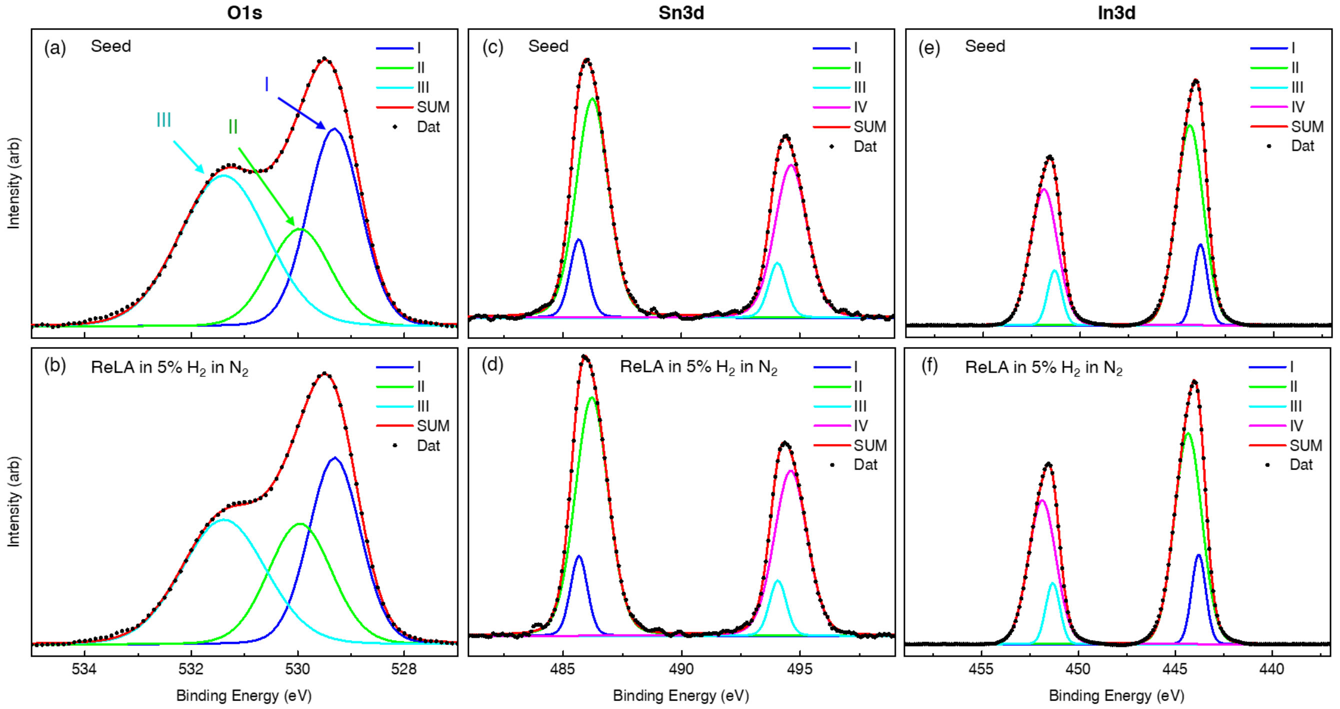

Figure S19. High resolution (a-b) Sn3d, (c-d) O1s and (e-f) In3d XPS spectra (black dots) for the seed ITO films before and after laser processing with a single pulse at 125 $mJcm^{-2}$ into an ambient atmosphere of 5% H_2 in N_2, respectively. The blue, green, cyan, and pink solid lines present the deconvoluted peaks for each core-level and the red solid line presents the cumulative fit to the data.

EDX data and analysis

The EDX images for the (a) seed ITO film and laser processed with a single 125 $mJcm^{-2}$ pulse in (b) 5% H_2 in N_2 and (c) O_2 are presented in Figure S20. Across the image, the average emitted X-ray intensity at the characteristic energy of each element within the sample is measured in order to build a depth profile of the relative elemental concentration across the film (Figure S21). To better compare the films, the intensity of each individual element is normalised to its peak value and the zero-point distance is set to the bottom of the film. This is done by setting the "bottom" of the film to the point where the normalised grey value intensity of In passes 0.2. This value is chosen as it gives a reasonable film bottom that allows each film to align. The normalised grey value intensities for (a) tin, (b) oxygen, (c) indium, (d) platinum and (e) silicon from the defined "bottom" of the seed (grey squares) ITO film and those subject to ReLA at 125 $mJcm^{-2}$ pulse in 5% H_2 in N_2 (red squares) and (c) O_2 (green squares) are presented in Figure S22.

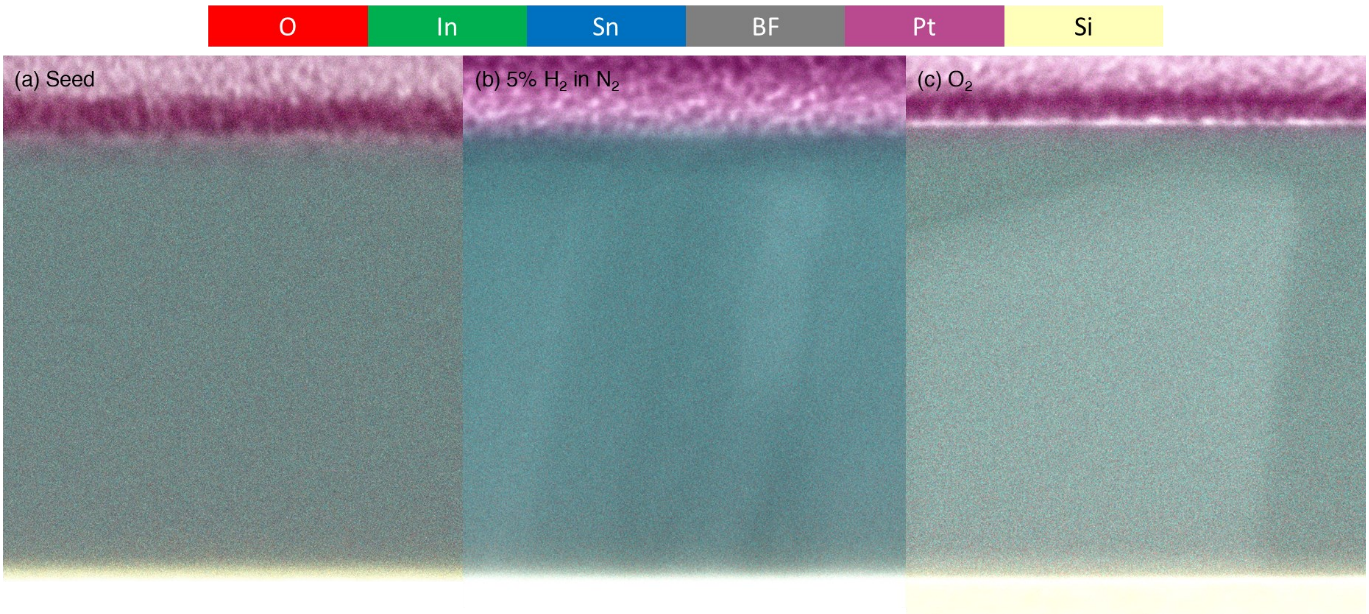

Figure S20. Transmission Electron Microscopy bright-field cross-sectional images (greyscale) of the (a) seed ITO film and those subject to single-pulse reactive laser annealing at 125 $mJcm^{-2}$ into a 100 psig pressurised environment of (b) 5% H_2 in N_2 and (c) 100% O_2. The brightfield image is overlayed with images of the detected characteristic X-ray intensity of oxygen (red), indium (green), tin (blue), platinum (purple), and silicon (yellow), as measured *via* energy-dispersive X-Ray spectroscopy.

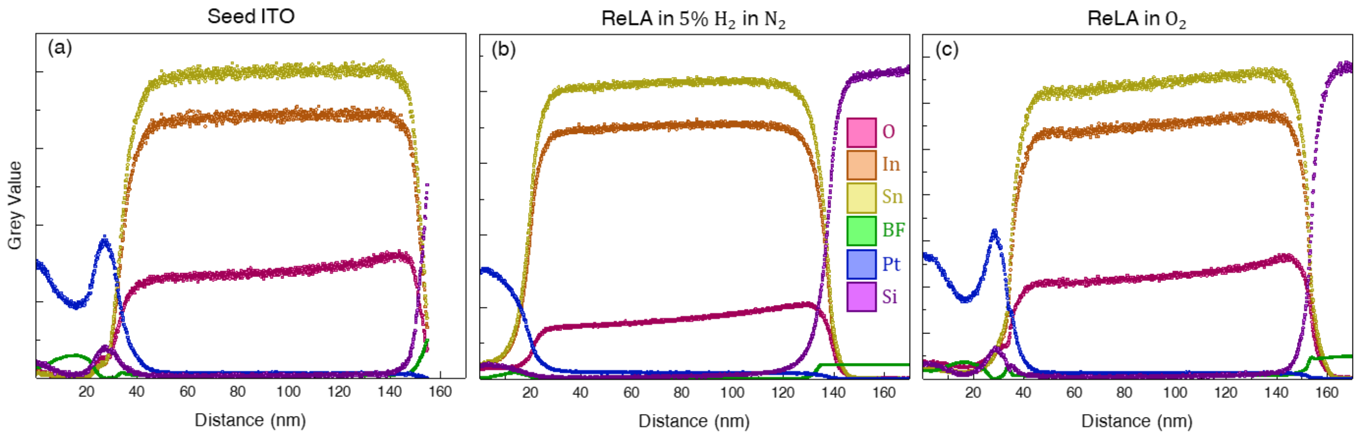

Figure S21. Depth-profiles of the energy-dispersive X-Ray spectroscopy intensity for the characteristic peaks of oxygen (pink), indium (orange), tin (yellow), platinum (blue), and silicon (purple) for the (a) seed ITO film and those subject to single-pulse reactive laser annealing at 125 $mJcm^{-2}$ into a 100 psig pressurised environment of (b) 5% H_2 in N_2 and (c) 100% O_2. The green squares present the depth profile of bright-field image greyscale intensity, for reference.

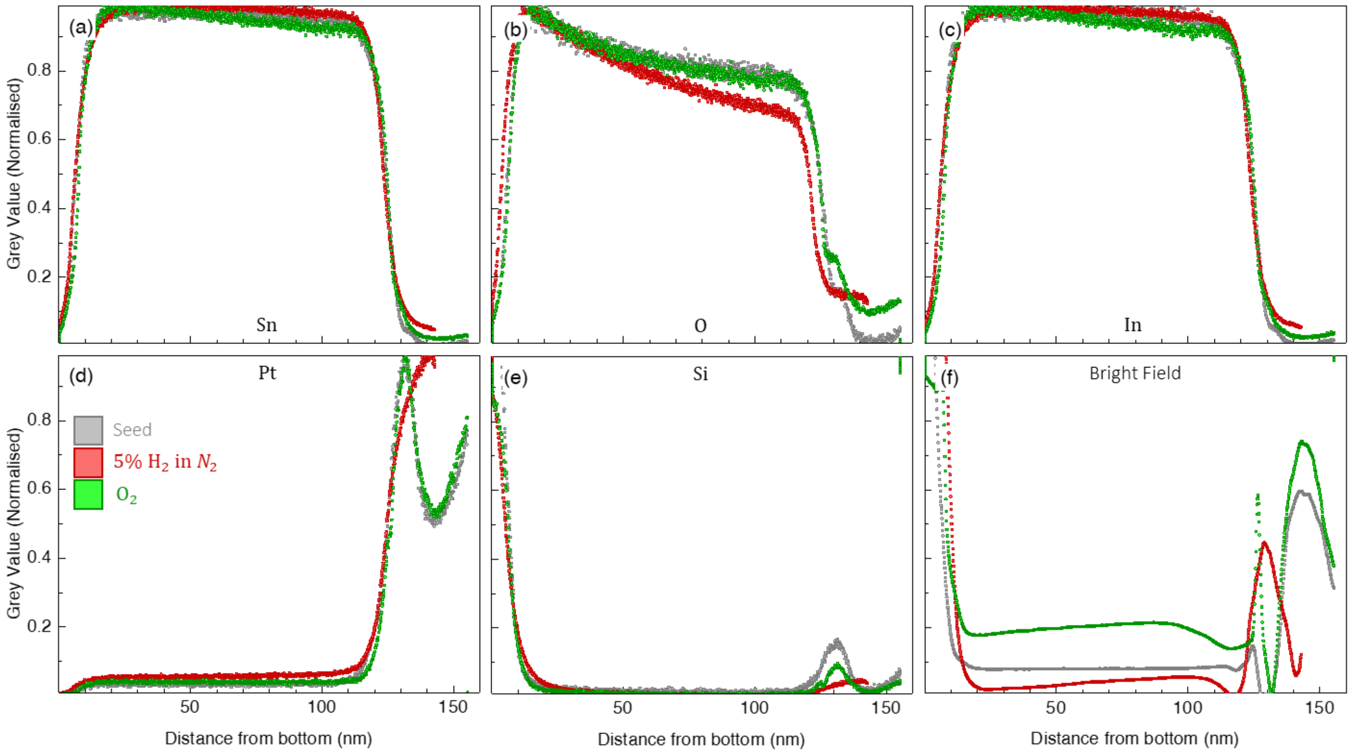

Figure S22. The normalised grey value intensities for (a) tin, (b) oxygen, (c) indium, (d) platinum and (e) silicon from the defined "bottom" of the seed (grey squares) ITO film and those subject to ReLA at 125 $mJcm^{-2}$ pulse in 5% H_2 in N_2 (red squares) and (c) O_2 (green squares). (f) Presents the depth profile of bright-field image greyscale intensity, for reference.

Section F: Opto-thermal simulations

This section contains the results of opto-thermal calculations, performed using a home-built code to elucidate the temperature rise across the structure at time, t, following the laser ignition. We do so by integrating the light propagation properties (optical constants of seed ITO, native SiO_2 and Si), in order to define the detailed spatial absorption distribution, A(z), as a function of the distance from the top surface, z. A(z) is presented in Figure S23.

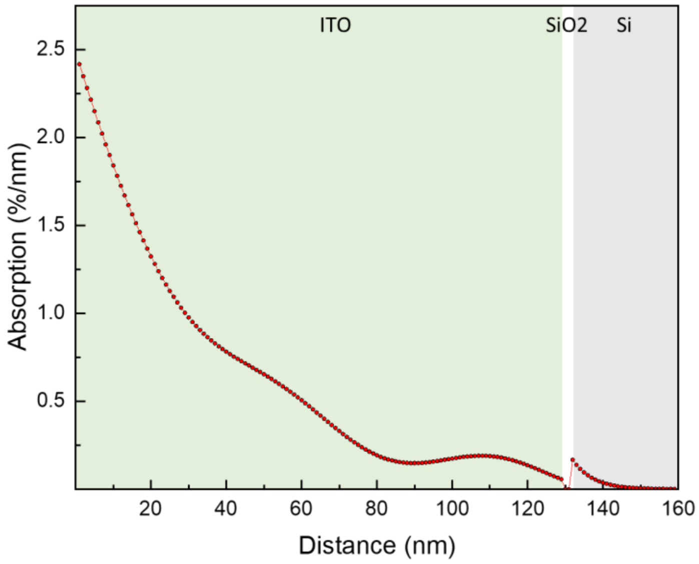

Figure S23. Absorption distribution (in % per nm) across the depth of the sample structure.

Combined with the temporal and spectral profile of the experimental laser (Figure S24a and Figure S24b, respectively), we are able to solve the one-dimensional (1D) heat transport equation to calculate the temperature transients at each point in the structure.

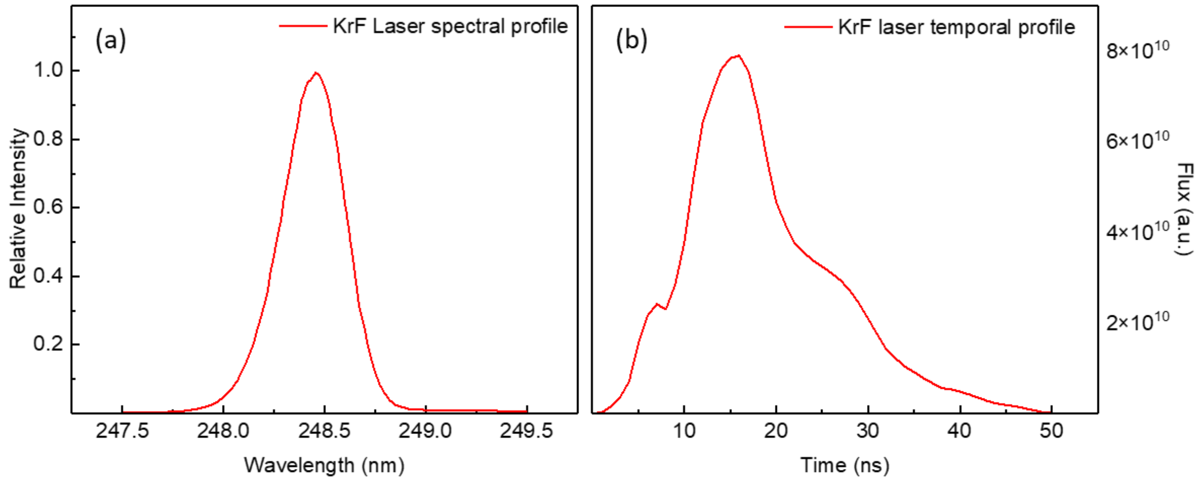

Figure S24. Experimental KrF laser (a) spectral and (b) temporal profile.

Specifically, the obtained absorption spatial distribution, A(z), and the experimental laser pulse profile, $\varphi(t)$, are used to determine the laser induced heating source, $Q(z,t) = A(z)\varphi(t)$, and solved numerically in the 1D heat diffusion equation:[12]

$$c(z)\rho(z)\frac{\partial T(z,t)}{\partial t} = \frac{\partial}{\partial z}\left[k(z)T(z,t)\frac{\partial T(z,t)}{\partial z}\right] + Q(z,t) \qquad \text{(S4)}$$

where T(z, t) is the local temperature transient and $\rho(z)$ is the mass density. c(z) is the heat capacity, k(z) is the thermal conductivity (both dependent on z due to the interchange between different materials).

The total laser fluence is given by:

$$f = \int_{-\infty}^{+\infty} \varphi(t)dt \tag{S5}$$

and is set here to match the experimental value of $125\ \mathrm{mJcm^{-2}}$. For simplicity, we assume a linear (*i.e.,* temperature independent) response. In Figure S25a, we present the temperature transient, $\Delta T(t)$, in Kelvin at the top (red line) and bottom (blue line) of the ITO film. We used materials properties from literature, as summarised in Table S4.

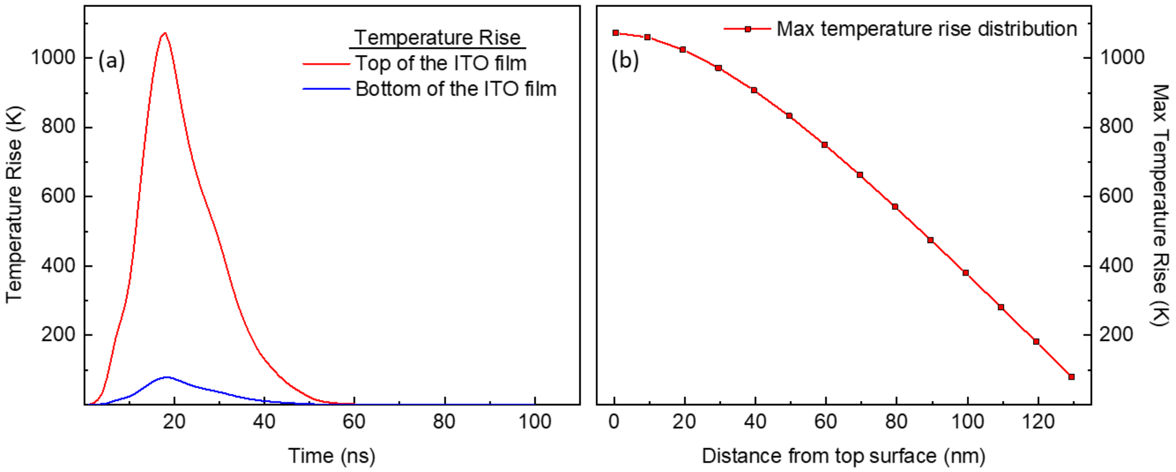

Figure S25. (a) Temperature rise, $\Delta T(t)$, at the top (red line) and bottom (blue line) of the ITO film. (b) Peak normalized temperature gradient, $\Delta T(z)$, in the ITO film.

Table S4. Material properties of ITO, SiO_2, and Si used for the opto-thermal calculations.

	**ITO**	**SiO_2**	**Si**[13]
**Mas density (kg m^{-3})**	7180[14]	2650[15]	2330
**Heat Capacity (J Kg^{-1} K^{-1})**	340[13]	725[16]	710
**Thermal conductivity (W m^{-1} K^{-1})**	5.38[17]	1.4[15]	149

By studying the linear heat transport regime, one can get insights into the temperature transients and gradients that can be developed, even without considering nonlinear effects on the materials' properties, nor the thermodynamics of phase changes, *e.g.,* melting and re-solidification. Such a temperature gradient (only for the ITO layer) is presented in Figure S25b. The temperature rise at the top of the film is estimated to be ~1000 K, which should be more than sufficient to induce the mobility of oxygen throughout the lattice. Furthermore, we note that the peak temperature rise, ΔT, at the bottom of the film is ~ 80 degrees above room temperature (106.85 °C at the bottom of the film), verifying the 'cold' nature of the LA process and highlighting the applicability of the ReLA process for thin films on thermally sensitive substrates. A final note is that the 1D heat diffusion suffices to describe the temperature gradient in the case of thin films and substrates with high thermal conductivity. However, when lateral temperature distribution can no longer be ignored, the use of more sophisticated 2D or 3D models become more appropriate.

References

1. Fujiwara, H. & Kondo, M. Effects of carrier concentration on the dielectric function of ZnO:Ga and In2O3:Sn studied by spectroscopic ellipsometry: Analysis of free-carrier and band-edge absorption. *Physical Review B* **71**, 075109 (2005).

2. Kim, H. *et al.* Electrical, optical, and structural properties of indium–tin–oxide thin films for organic light-emitting devices. *Journal of Applied Physics* **86**, 6451–6461 (1999).

3. Ellmer, K. & Mientus, R. Carrier transport in polycrystalline ITO and ZnO:Al II: The influence of grain barriers and boundaries. *Thin Solid Films* **516**, 5829–5835 (2008).

4. Ellmer, K. & Mientus, R. Carrier transport in polycrystalline transparent conductive oxides: A comparative study of zinc oxide and indium oxide. *Thin Solid Films* **516**, 4620–4627 (2008).

5. Masetti, G., Severi, M. & Solmi, S. Modeling of carrier mobility against carrier concentration in arsenic-, phosphorus-, and boron-doped silicon. *IEEE Transactions on Electron Devices* **30**, 764–769 (1983).

6. Hillier, J. A. *et al.* When ellipsometry works best: A case study with transparent conductive oxides. *ACS Photonics* **7**, (2020).

7. Hilfiker, J. N. & Tompkins, H. G. *Spectroscopic Ellipsometry: Practical Application To Thin Film Characterization*. (Momentum Press, 2015).

8. Jellison, G. E. & Modine, F. A. Parameterization of the optical functions of amorphous materials in the interband region. *Applied Physics Letters* **69**, 371–373 (1996).

9. de Sousa Meneses, D., Malki, M. & Echegut, P. Structure and lattice dynamics of binary lead silicate glasses investigated by infrared spectroscopy. *Journal of Non-Crystalline Solids* **352**, 769–776 (2006).

10. Uprety, P., Junda, M. M., Salmon, H. & Podraza, N. J. Understanding near infrared absorption in tin doped indium oxide thin films. *Journal of Physics D: Applied Physics* **51**, 295302 (2018).

11. Gonçalves, G. *et al.* Influence of post-annealing temperature on the properties exhibited by ITO, IZO and GZO thin films. *Thin Solid Films* **515**, 8562–8566 (2007).

12. Yarali, E. *et al.* Recent Progress in Photonic Processing of Metal-Oxide Transistors. *Advanced Functional Materials* **30**, 1906022 (2020).

13. Wang, L., Wen, J., Yang, C. & Xiong, B. Potential of ITO thin film for electrical probe memory applications. *Science and Technology of Advanced Materials* **19**, 791–801 (2018).

14. Schneider, S. J. *Journal of Research of the National Bureau of Standards, Section A: Physics and Chemistry*. vol. 65A (The Bureau, 1961).

15. Grove, A. S. *Physics and Technology of Semiconductor Devices*. Wiley 1991.

16. Andersson, S. & Dzhavadov, L. Thermal conductivity and heat capacity of amorphous SiO_2: pressure and volume dependence. *Journal of Physics: Condensed Matter* **4**, 6209–6216 (1992).

17. Ashida, T. *et al.* Thermal transport properties of polycrystalline tin-doped indium oxide films. *Journal of Applied Physics* **105**, 073709 (2009).
